# An approach to unified formulae for likelihood ratio calculation in pairwise kinship analysis

**DOI:** 10.3389/fgene.2024.1226228

**Published:** 2024-02-07

**Authors:** Guanju Ma, Qian Wang, Bin Cong, Shujin Li

**Affiliations:** ^1^ Hebei Key Laboratory of Forensic Medicine, Research Unit of Digestive Tract Microecosystem Pharmacology and Toxicology, Chinese Academy of Medical Sciences, College of Forensic Medicine, Hebei Medical University, Shijiazhuang, China; ^2^ Hainan Tropical Forensic Medicine Academician Workstation, Haikou, China

**Keywords:** pairwise kinship testing, likelihood ratio, unified formulae, divide-and-conquer method, R package construction

## Abstract

**Introduction:** The likelihood ratio (LR) can be an efficient means of distinguishing various relationships in forensic fields. However, traditional list-based methods for derivation and presentation of LRs in distant or complex relationships hinder code editing and software programming. This paper proposes an approach for a unified formula for LRs, in which differences in participants’ genotype combinations can be ignored for specific identification. This formula could reduce the difficulty of by-hand coding, as well as running time of large-sample-size simulation.

**Methods:** The approach is first applied to a problem of kinship identification in which at least one of the participants is alleged to be inbred. This can be divided into two parts: i) the probability of different identical by descent (IBD) states according to the alleged kinship; and ii) the ratio of the probability that specific genotype combination can be detected assuming the alleged kinship exists between the two participants to the similar probability assuming that they are unrelated, for each state. For the probability, there are usually recognized results for common identification purposes. For the ratio, subscript letters representing IBD alleles of individual A’s alleles are used to eliminate differences in genotype combinations between the two individuals and to obtain a unified formula for the ratio in each state. The unification is further simplified for identification cases in which it is alleged that both of the participants are outbred. Verification is performed to show that the results obtained with the unified and list-form formulae are equivalent.

**Results:** A series of unified formulae are derived for different identification purposes, based on which an R package named KINSIMU has been developed and evaluated for use in large-size simulations for kinship analysis. Comparison between the package with two existing tools indicated that the unified approach presented here is more convenient and time-saving with respect to the coding process for computer applications compared with the list-based approach, despite appearing more complicated. Moreover, the method of derivation could be extended to other identification problems, such as those with different hypothesis sets or those involving multiple individuals.

**Conclusion:** The unified approach of LR calculation can be beneficial in kinship identification field.

## 1 Introduction

In kinship identification or forensic genealogy, at least two alleged relationships between participants need to be confirmed or excluded using the genetic information available. However, confirmation or exclusion can be difficult, as the alleged relationships can be much more complicated than those in well-researched parentage cases. With the development of forensic databases and sequencing technologies, increasingly complex kinship relationships need to be studied in forensic genetics and especially forensic genealogy. Several approaches can be used for identification of these complicated relationships, including the likelihood ratio (LR) method or simple application of an identity by state (IBS) score. For distant kinship identification in forensic genealogy, it has been claimed that “the traditional LR approach as a single source of classification is as good as, and in some cases even better than, the alternative approaches” (Kling and Tillmar, 2019).

In order to conduct complex kinship identification, it is essential to evaluate the practicality of specific panels or marker types (Mo et al., 2018; Li et al., 2019; Liu et al., 2020; Wu et al., 2021; Zhao et al., 2021; Du et al., 2023). Such assessments often necessitate pedigree investigations based on real cases or computer simulations. As kinship complexity increases, so too does the difficulty of investigating real cases: either the target cases are rare in the population (such as inbreeding cases); or the confirmation of the participants is challenging (for example, to confirm the relationship between a pair of first cousins, their common grandparents, and their parents who are full-siblings to each other should also be detected), making simulation a more realistic method. There are several application scenarios for simulation methods in this field, including i) assessing the sensitivity and specificity of specific markers in identification (Phillips et al., 2012; Mo et al., 2018; Liu et al., 2020; Du et al., 2023); ii) comparing different parameters in the same identification based on the same panel, e.g., LR calculated based on length information vs LR calculated based on length + sequence information (Staadig and Tillmar, 2019; Liu et al., 2020); iii) estimating the required number of markers in specific identification using curve fitting, through a quantity of simulation based on different subsets of current loci combinations, when the current combination cannot meet the identification requirements (Mo et al., 2016; Du et al., 2023).

Certain obstacles may be encountered when using the LR method in large-sample-size simulation, owing to the presentation of calculation results and the coding logic based on such results in various studies. In such studies, LR are presented as the listings of all possible genotype combinations of participants, followed by the application of different formulae in different cases. This necessitates dividing the calculation of LR into multiple types and using multi-layer logical comparison functions, such as “if (if (…))”, during the coding process. Although current tools can eliminate the need for users to carry out the complex coding processes mentioned earlier, in research situations where such tools are not available (such as rare complex kinship cases), users still need to compute and simulate for themselves. Thus, it would be beneficial to establish a unified formula for distant or complex kinship identification that disregards the participants’ genotype combinations and avoids logical comparison functions as much as possible in the coding process, mitigating the difficulty of manual coding and the time required. Egeland *et al.* devised a unified formula for pairwise non-inbred kinship testing in Egeland et al. (2017), which was used in *Familias* 3.0 (Egeland et al., 2016).

This paper presents an alternative approach that delivers equivalent results to Egeland’s formula, but can be easily extended to inbreeding relationships, owing to its concise derivation methodology. A package named *KINSIMU* containing a series of newly defined functions for the R platform is provided, based on the unified formula, and can be used for large-sample-size simulation/calculation in specific kinship analysis based on independent genetic markers.

## 2 Methods and results

### 2.1 General setting

In pairwise kinship identification, there is no detectable genetic information other than the genotype combination of the two participants, who are labeled as individual A and B, respectively, in this paper. Suppose that the detected genotypes of them are ***ab*** and ***cd***, respectively, where the four alleles can be identical to each other or not. An individual is called “inbred” if his/her parents are biological relatives, or “outbred” otherwise. Mutation is only considered when constructing the paternity index calculation function in the construction of *KINSIMU* package and not in the inference process in this section, which will be discussed in section 3.

In the derivation, some symbols with specific meanings will appear, including:

I) In this article, the identity symbol “≡” is utilized to denote the identity between particular alleles or genotypes, and this will occur in two situations:
  i) It is preceded by a capital letter and followed by two lowercase letters, indicating the event that “the two alleles of the individual represented by the capital letter are those represented by these two lowercase letters”;
  ii) Letters on both side of it are lowercase, meaning that the alleles or genotypes on both sides are identical to each other;

It should be noted that the “identity” status represented by the symbol can arise from genetic inheritance or by random occurrence.

II) The symbol ***p*** with subscript lowercase letter such as “*p*_*c*_” denotes the frequency of allele represented by the subscript letter;
III) The symbol 1 with two subscript lowercase letters equals to 1 if the two alleles represented by the two letters are identical and to 0 otherwise. For instance, $1_{ac}$ equals to 1 only if *a* ≡ *c*.
IV) Lowercase letters with subscript “***I***” such as “*a*_*I*_” means the allele identical by descent (IBD) to the corresponding allele. If there is no other information, the probability of an IBD allele being identical to a detected allele equals to the corresponding 1 parameter, e.g., $\mathrm{Pr}\left(a_{I}\equiv c\right)=1_{ac}$ ;
V) The symbol “***x***_***I***_” and “***y***_***I***_” represent the alleles not IBD to none of the detected alleles of participants other than individual B, where “*x*_*I*_” is unrelated to “*y*_*I*_”. Without other information, the probability of these alleles being a specific allele in individual B’s genotype equals to the corresponding *p* parameter, e.g., $\mathrm{Pr}\left(x_{I}\equiv c\right)=p_{c}$ .
VI) The symbol ***d*** with a subscript, which is formed by a capital letter stand for a specific individual followed by a lowercase letter denotes specific allele, such as ***d***_***Ac***_, denotes the dosage of the corresponding allele in the genotype of the corresponding individual, e.g., $d_{Ac}=1_{ac}+1_{bc}$ if the genotype of individual A is *ab.*

### 2.2 Overall deduction of unified LR formulae in pairwise identification

As discussed in multiple articles (Gjertson et al., 2007; Egeland and Sheehan, 2008; Skare et al., 2009; Egeland et al., 2017; Slooten, 2020), in pairwise kinship analysis, the probabilities of different relationships existing between participants can be evaluated conditional on the genetic evidence, i.e., $\mathrm{Pr}\left(H\left|E\right.\right)$ , with terms *H* and *E* denoting the alleged hypothesis and the observed genetic evidence, respectively. It is difficult to calculate such probabilities directly; however, the ratio of different incompatible hypotheses can be computed according to Bayes’ rule:

$$\frac{\mathrm{Pr}\left(H_{p}\left|E\right.\right)}{\mathrm{Pr}\left(H_{d}\left|E\right.\right)}=\frac{\mathrm{Pr}\left(H_{p}\right)}{\mathrm{Pr}\left(H_{d}\right)}\times \frac{\mathrm{Pr}\left(E\left|H_{p}\right.\right)}{\mathrm{Pr}\left(E\left|H_{d}\right.\right)}$$ (1)

In the above equation, terms *H*_*p*_ (Plaintiff’s Hypothesis) and *H*_*d*_ (Defendant’s hypothesis) denote the two hypotheses being compared. For all of the inference process in this paper, we define *H*_*d*_ as the hypothesis that “the two individuals are both outbred and unrelated to each other”. Meanwhile, the definition of *H*_*p*_ varies depending on the scenario, such as “individual A is outbred and the biological father of outbred individual B” in paternity testing. In the kinship identification field, the LR is defined as the ratio of conditional probabilities in the formula:

$$LR=\frac{\mathrm{Pr}\left(E\left|H_{p}\right.\right)}{\mathrm{Pr}\left(E\left|H_{d}\right.\right)}$$ (2)

The ratio $\mathrm{Pr}\left(H_{p}\right)/\mathrm{Pr}\left(H_{d}\right)$ , or *π*_1_/*π*_0_ in several articles (Gjertson et al., 2007; Egeland and Sheehan, 2008), is called the prior odds, representing how much more likely *H*_*p*_ is to be true than *H*_*d*_ without the genetic data *E*. If the prior odds are considered to be 1, i.e., the two hypotheses are of equal probabilities without the genetic information, which is the most commonly used assumption in forensic practice (Slooten, 2020), then LR can represent the ratio of likelihood initially required in Eq. 1.

**FIGURE 1:**
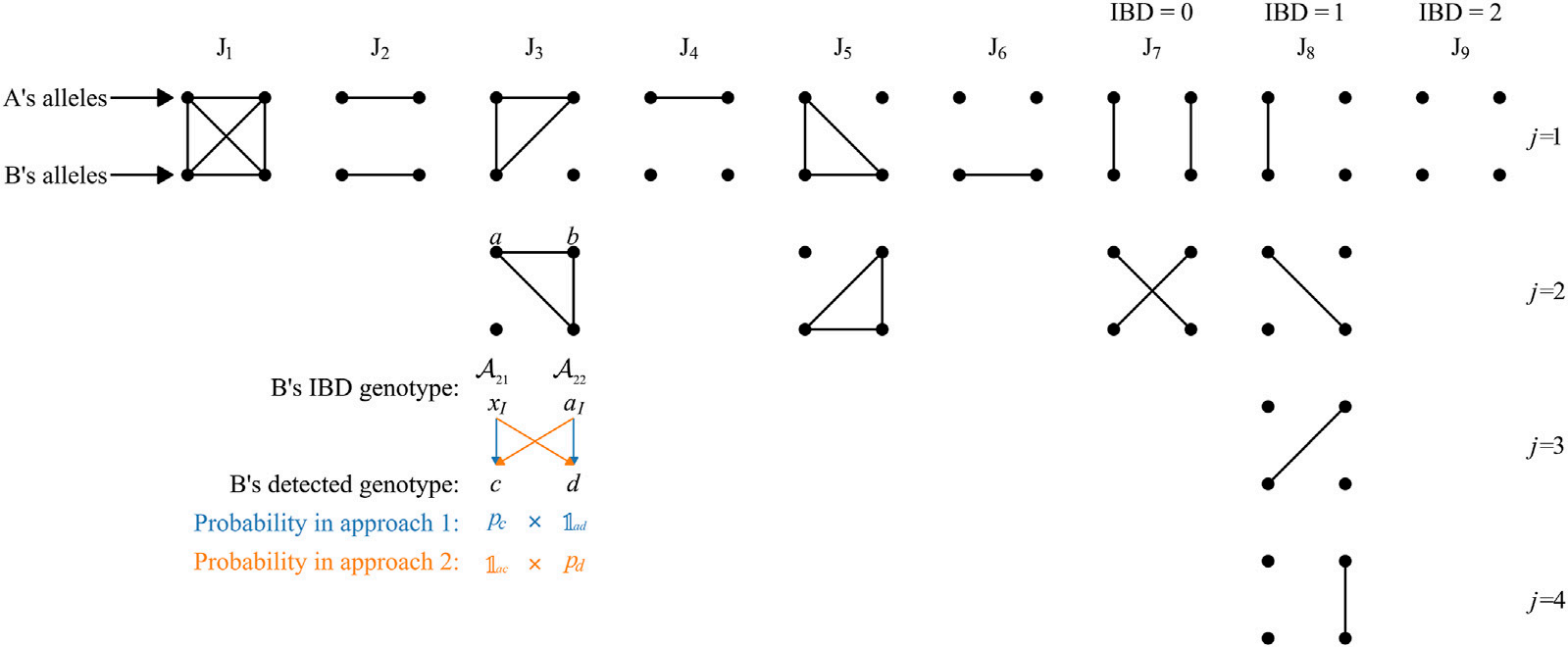
Nine Jacquard states considering inbreeding. The figure is modified from Figure 1 of Brustad et al. (2021b). Each group of 2 × 2 dots represents a pair of participants, each row of two dots represents the two alleles of an individual, and IBD alleles are connected by lines. The states *J*_9_, *J*_8_, and *J*_7_ do not involve inbreeding and are sometimes denoted IBD = 0, 1, and 2, respectively. The third group of dots in *J*_8_ column was mistakenly drawn as the second in *J*_7_ column in Brustad et al. (2021b); the mistake is corrected in this figure.

Further derivation can be made from Eq. 2 considering the fact that *H*_*p*_ can be further divided into several multiple exclusive states according to whether the four alleles of individual A and B are IBD to each other, such as the nine Jacquard states in inbreeding identification (Jacquard, 1972) (*J*_1_ → *J*_9_, see Figure 1 of (Brustad et al., 2021b), which is reproduced with modification as Figure 1 in this work) or the three IBD states commonly be used in non-inbred ones (Egeland et al., 2017). However, there can be only one such state, i.e., *J*_9_ or *IBD* = 0, under the *H*_*d*_ set, as in section 2.1. Thus, LR can be calculated as Eq. 3.

$$LR=\left\{\begin{array}{c} \frac{\overset{9}{\underset{i=1}{\sum }}\left[\mathrm{Pr}\left(J_{i}\left|H_{p}\right.\right)\times \mathrm{Pr}\left(E\left|J_{i},H_{p}\right.\right)\right]}{\mathrm{Pr}\left(E\left|J_{9},H_{d}\right.\right)}\\ \frac{\overset{2}{\underset{i=0}{\sum }}\left[\mathrm{Pr}\left(IBD=i\left|H_{p}\right.\right)\times \mathrm{Pr}\left(E\left|IBD=i,H_{p}\right.\right)\right]}{\mathrm{Pr}\left(E\left|IBD=0,H_{d}\right.\right)} \end{array}\right.$$ (3)

If a specific state is set, the probability that *E* happens is fixed, irrespective of the hypothesis. Thus, LR can be calculated with Eq. 4, where Δ_*i*_ denotes the *i*th Jacquard coefficient (Brustad et al., 2021b) under *H*_*p*_, equal to the probability that *J*_*i*_ happens between two individuals with specific relationship in the absence of their genetic information, i.e., $\mathrm{Pr}\left(J_{i}\left|H_{p}\right.\right)$ ; and *κ*_*i*_ represents the IBD coefficient (Egeland et al., 2017), equal to the similar probability under non-inbred assumption, $\mathrm{Pr}\left(IBD=i\left|H_{p}\right.\right)$ .

$$LR=\left\{\begin{array}{c} \overset{9}{\underset{i=1}{\sum }}\left[\Delta_{i}\times \frac{\mathrm{Pr}\left(E\left|J_{i}\right.\right)}{\mathrm{Pr}\left(E\left|J_{9}\right.\right)}\right]\\ \overset{2}{\underset{i=0}{\sum }}\left[\kappa_{i}\times \frac{\mathrm{Pr}\left(E\left|IBD=i\right.\right)}{\mathrm{Pr}\left(E\left|IBD=0\right.\right)}\right] \end{array}\right.$$ (4)

Therefore, the unification of LR can be divided into two problems: the calculation of the ratio of conditional probabilities under each state according to the participants’ genotypes (as discussed in section 2.3); and the Δ/*κ* distribution according to the alleged relationship (Pinto et al., 2010), which remains unchanged if the identification purpose is set (e.g., $\kappa =\left\{1/4,1/2,1/4\right\}$ for full-sibling identification) and can be obtained from the pedigree tree using existing tools (Vigeland, 2022). Thus, it is not necessary to infer the distribution of the two types of coefficients per case if there is a recognized result for the target identification.

### 2.3 Unification of the ratio of conditional probabilities under each state

#### 2.3.1 When at least one of the two individuals is inbred

LR calculation when at least one of the two individuals is inbred has been well discussed in previous publications (Jacquard, 1972; Brustad et al., 2021a; b), and a method has been developed considering the Jacquard states. The probability $\mathrm{Pr}\left(E\left|J_{i}\right.\right)$ can be listed in a 9 × 9 table, as in Table 1 in (Brustad et al., 2021b). Herein, we improve the method by eliminating the difference between the genotype combinations of the two individuals, i.e., “*E*”; thus, the probability ratio $\left.\mathrm{Pr}\left(E\left|J_{i}\right.\right)/\mathrm{Pr}\left(E\left|J_{9}\right.\right)\right.$ can be listed in a 1 × 9 table, and LR can be calculated with a unified formula for specific identification. The ratio can be further deducted based on the fact that the event *E* can be understood as the event that the following two events happened simultaneously: *A* ≡ *ab* and *B* ≡ *cd*:

$$\frac{\mathrm{Pr}\left(E\left|J_{i}\right.\right)}{\mathrm{Pr}\left(E\left|J_{9}\right.\right)}=\frac{\mathrm{Pr}\left(A\equiv ab,B\equiv cd\left|J_{i}\right.\right)}{\mathrm{Pr}\left(A\equiv ab,B\equiv cd\left|J_{9}\right.\right)}=\frac{\mathrm{Pr}\left(A\equiv ab\left|J_{i}\right.\right)}{\mathrm{Pr}\left(A\equiv ab\left|J_{9}\right.\right)}\times \frac{\mathrm{Pr}\left(B\equiv cd\left|A\equiv ab,J_{i}\right.\right)}{\mathrm{Pr}\left(B\equiv cd\left|A\equiv ab,J_{9}\right.\right)}$$ (5)

The former ratio in Eq. 5 can be calculated differently depending on whether the two alleles of individual A are IBD. If the two alleles of individual A are not IBD to each other and no other information is considered, the probability that the individual’s genotype is *ab* should equal the frequency of the genotype, i.e., $\left.\mathrm{Pr}\left(A\equiv ab\left|J_{i}\right.\right)/\mathrm{Pr}\left(A\equiv ab\left|J_{9}\right.\right)=1\right.$ when $i\in \left[5,9\right]$ . Otherwise, if the two alleles of individual A are IBD to each other, and mutation is not considered, the probability of individual A being a specific genotype equals *p*_*a*_ when *a* ≡ *b* and 0 otherwise, i.e., $\mathrm{Pr}\left(A\equiv ab\left|J_{i}\right.\right)=1_{ab}p_{a}$ when $i\in \left[1,4\right]$ . Thus, the former ratio of Eq. 5 can be calculated as Eq. 6 when *i* ∈ [1, 4].

$$\frac{\mathrm{Pr}\left(A\equiv ab\left|J_{1\to 4}\right.\right)}{\mathrm{Pr}\left(A\equiv ab\left|J_{9}\right.\right)}=\left\{\begin{array}{cccccc} & \frac{1_{ab}p_{a}}{p_{a}^{2}} & & , & & a\equiv b\\ & 0 & & , & & a\neq b \end{array}\right\}=\frac{1_{ab}}{p_{a}}$$ (6)

In summary, if consider all Jacquard states, the former ratio of Eq. 5 can be calculated as Eq. 7.

$$\frac{\mathrm{Pr}\left(A\equiv ab\left|J_{i}\right.\right)}{\mathrm{Pr}\left(A\equiv ab\left|J_{9}\right.\right)}=\left\{\begin{array}{ccccc} \frac{1_{ab}}{p_{a}} & & , & & i\in \left[1,4\right]\\ 1 & & , & & i\in \left[5,9\right] \end{array}\right.$$ (7)

The latter ratio in Eq. 5 can be derived with individual B’s genotype from the perspective of individual A’s IBD alleles (defined as “IBD genotype” in this work). Under each Jacquard state, the IBD genotype of individual B should be set, there is only one possible type when *i* = 1, 2, 4, 6, or 9, e.g., it must be *a*_*I*_ *a*_*I*_ under *J*_1_; otherwise, there can be multiple possible types under a same state, e.g., *a*_*I*_ *x*_*I*_ or *x*_*I*_ *a*_*I*_ under *J*_3_ (see Figure 1). If we set “ $G_{j}$ ” as individual B’s *j*th possible IBD genotypes under a specific Jacquard state. It can be derived according to the Law of Total Probability that,

$$\mathrm{Pr}\left(B\equiv cd\left|A\equiv ab,J_{i}\right.\right)=\underset{j}{\sum }[\mathrm{Pr}\left(B\equiv G_{j}\left|A\equiv ab,J_{i}\right.\right)\times \mathrm{Pr}\left(B\equiv cd\left|B\equiv G_{j},A\equiv ab,J_{i}\right.\right)]$$ (8)

where $\mathrm{Pr}\left(B\equiv G_{j}\left|A\equiv ab,J_{i}\right.\right)$ denotes the probability that individual B’s IBD genotype is $G_{j}$ under the corresponding state before detecting his/her actual genotype. It can be seen that, if there are multiple possible types of such IBD genotype, this probability should be equivalent for each type owing to the absence of other genetic information, i.e., Eq. 8 can be further derived as Eq. 9.

$$\mathrm{Pr}\left(B\equiv G_{j}\left|A\equiv ab,J_{i}\right.\right)=\left\{\begin{array}{ccccc} & 1 & & , & i\in \left\{1,2,4,6,9\right\}\\ \frac{1}{2} & & , & & i\in \left\{3,5,7\right\}\\ \frac{1}{4} & & , & & i=8 \end{array}\right.$$ (9)

And the probability $\mathrm{Pr}\left(B\equiv cd\left|B\equiv G_{j},A\equiv ab,J_{i}\right.\right)$ stands for the probability that individual B’s actual genotype is detected as *cd* given that his/her IBD genotype is $G_{j}$ . If the IBD genotype is set, this probability should be fixed regardless of other conditions, i.e.,

$$\mathrm{Pr}\left(B\equiv cd\left|B\equiv G_{j},A\equiv ab,J_{i}\right.\right)=\mathrm{Pr}\left(G_{j}\equiv cd\right)$$ (10)

If defined $A_{j1}$ and $A_{j2}$ as the first and the second allele of $G_{j}$ , there are two exclusive approach for IBD genotype $G_{j}$ to be detected as *cd* when *c* ≠ *d* and not considering mutation: **i)** $A_{j_{1}}\equiv c,\;A_{j_{2}}\equiv d$ , and **ii)** $A_{j_{1}}\equiv d,\;A_{j_{2}}\equiv c$ . Thus, according to the Rule of total probability,

$$\mathrm{Pr}\left(G_{j}\equiv cd\right)=\mathrm{Pr}\left(A_{j_{1}}\equiv c\right)\times \mathrm{Pr}\left(A_{j_{2}}\equiv d\left|A_{j_{1}}\equiv c\right.\right)+\mathrm{Pr}\left(A_{j_{1}}\equiv d\right)\times \mathrm{Pr}\left(A_{j_{2}}\equiv c\left|A_{j_{1}}\equiv d\right.\right)$$ (11)

As discussed in section 2.1, the probabilities $\mathrm{Pr}\left(A_{j_{1}}\equiv c\right)$ and $\mathrm{Pr}\left(A_{j_{1}}\equiv d\right)$ equal the corresponding 1 parameter if $A_{j_{1}}$ is IBD to one of individual A’s alleles, or the corresponding ***p*** parameter when $A_{j_{1}}\equiv x_{I}$ . The probabilities $\mathrm{Pr}\left(A_{j_{2}}\equiv d\left|A_{j_{1}}\equiv c\right.\right)$ and $\mathrm{Pr}\left(A_{j_{2}}\equiv c\left|A_{j_{1}}\equiv d\right.\right)$ equal $1_{cd}$ if the two alleles $A_{j_{1}}$ and $A_{j_{1}}$ are IBD to each other (under *J*_1,2,5,6_), otherwise, the probability can be calculated similarly to the former two probabilities, i.e.,

$$\mathrm{Pr}\left(A_{j_{2}}\equiv d\left|A_{j_{1}}\equiv c\right.\right)=\left\{\begin{array}{ccccc} 1_{cd} & & , & & J_{1},J_{2},J_{5},J_{6}\\ \mathrm{Pr}\left(A_{j_{2}}\equiv d\right) & & , & & other \end{array}\right.$$ (12)

When *c* ≡ *d*, the calculation result obtained with the above equation would be double of the actual probability, and the latter ratio in Eq. 5 would remain unchanged.

Taking the situation *J*_3_ as an example, under this state, one of individual B’s allele is IBD to both of individual A’s while the other one is not IBD to none of A’s alleles. As mentioned above, there can be two possible $G_{j}$ , i.e., *a*_*I*_ *x*_*I*_ (the first row of the third column in Figure 1) or *x*_*I*_ *a*_*I*_ (the second row). The two possible approaches of the second type of IBD genotype to be detected as *cd* are listed with blue and orange colors in Figure 1 that,

$$\mathrm{Pr}\left(G_{j}\equiv cd\right)=\mathrm{Pr}\left(x_{I}a_{I}\equiv cd\right)=p_{c}1_{ad}+1_{ac}p_{d}$$ (13)

Using a similar derivation, the same result can be achieved for the first type of IBD genotype, i.e., $\mathrm{Pr}\left(E\left|J_{3}\right.\right)=p_{c}1_{ad}+1_{ac}p_{d}$ . And for *J*_9_, $\mathrm{Pr}\left(G_{j}\equiv cd\right)=2p_{c}p_{d}$ . Thus,

$$\frac{\mathrm{Pr}\left(E\left|J_{3}\right.\right)}{\mathrm{Pr}\left(E\left|J_{9}\right.\right)}=\frac{1_{ab}}{p_{a}}\times \left(\frac{1_{ad}}{2p_{d}}+\frac{1_{ac}}{2p_{c}}\right)=\frac{1_{ab}}{p_{a}}\times \left(\frac{1_{ad}}{2p_{a}}+\frac{1_{ac}}{2p_{a}}\right)=\frac{1_{ab}\left(1_{ac}+1_{ad}\right)}{2p_{a}^{2}}$$ (14)

The detailed inference process for all states is shown in Section 1 of File S1 in Supplementary Materials, and the LR calculation in such cases can be unified as Eq. 15:

$$\begin{array}{cc} LR= & \frac{\Delta_{1}1_{ab}1_{ac}1_{cd}}{p_{a}^{3}}+\frac{\Delta_{2}1_{ab}1_{cd}}{p_{a}p_{c}}+\frac{\Delta_{3}1_{ab}\left(1_{ac}+1_{ad}\right)}{2p_{a}^{2}}+\frac{\Delta_{4}1_{ab}}{p_{a}}+\frac{\Delta_{5}1_{cd}\left(1_{ac}+1_{bc}\right)}{2p_{c}^{2}}\\ & +\frac{\Delta_{6}1_{cd}}{p_{c}}+\frac{\Delta_{7}\left(1_{ac}1_{bd}+1_{ad}1_{bc}\right)}{2p_{c}p_{d}}+\frac{\Delta_{8}\left[\left(1_{ac}+1_{bc}\right)p_{d}+\left(1_{ad}+1_{bd}\right)p_{c}\right]}{4p_{c}p_{d}}+\Delta_{9} \end{array}$$ (15)

Therefore, 9 elements (*p*_*a*_, *p*_*c*_, *p*_*d*_, $1_{ab}$ , $1_{cd}$ , $1_{ac}$ , $1_{ad}$ , $1_{bc}$ , and $1_{bd}$ ) need to be calculated in the determination of LR, which can be calculated uniformly based on the genotype data of the two individuals and brought into a unified formula.

#### 2.3.2 When both individual A and B are outbred

If an individual is outbred, his or her two alleles should not be IBD alleles, i.e., there is no possibility of one of Jacquard states *J*_1_ to *J*_6_ occurring when the two participants are both outbred. As discussed above, if the genotype $G_{j}$ is set, the probability that it is *cd* is independent of the hypothesis; thus, the calculation of the conditional probabilities’ ratios under *J*_7_–*J*_9_, i.e., the ratios when *IBD* = 2, 1, or 0, is the same, and the LR calculation can be simplified as follows:

$$LR=\frac{\kappa_{2}\left(1_{ac}1_{bd}+1_{ad}1_{bc}\right)}{2p_{c}p_{d}}+\frac{\kappa_{1}\left[\left(1_{ac}+1_{bc}\right)p_{d}+\left(1_{ad}+1_{bd}\right)p_{c}\right]}{4p_{c}p_{d}}+\kappa_{0}$$ (16)

Here, the number of elements needed in the unified calculation would be reduced to 6 (the calculation of *p*_*a*_, $1_{ab}$ and $1_{cd}$ are not needed). Furthermore, for identification where *κ*_2_ = 0, if we define *d*_*Ac*_ and *d*_*Ad*_ as section 2.1, Eq. 16 can be further simplified as follows and the number of elements needed would be reduced to 4 (*d*_*Ac*_, *d*_*Ad*_, *p*_*c*_ and *p*_*d*_):

$$LR=\frac{\kappa_{1}}{4}\left(\frac{d_{Ac}}{p_{c}}+\frac{d_{Ad}}{p_{d}}\right)+\kappa_{0}$$ (17)

#### 2.3.3 LR when *H*_*d*_ set as section 2.1 is ruled out in advance

Unified formulae can be applied even if *H*_*d*_, i.e., “both individual A and B are outbred and unrelated to each other” is ruled out in advance and the identification takes place between two hypotheses *H*_*p*_ and $H_{d}^{\prime }$ , under each of which the two individuals are relatives. For that case, LR can be calculated as the ratio of the two LRs with $\mathrm{Pr}\left(E\left|H_{p}\right.\right)$ and $\mathrm{Pr}\left(E\left|H_{d}^{\prime }\right.\right)$ being the numerators, i.e.,

$${LR}_{H_{p},H_{d}^{\prime }}\left(E\right)=\frac{{LR}_{H_{p},H_{d}}\left(E\right)}{{LR}_{H_{d}^{\prime },H_{d}}\left(E\right)}$$ (18)

For example, in father–daughter incest cases, if the mother–offspring relationship is confirmed and the genetic information of the alleged father is unavailable, an LR called the incest index (II) (Wenk et al., 1994; Wenk, 2007) can be calculated according to the genetic information of the mother–offspring pair to measure the probability of the incest event. For this index, *H*_*p*_ and $H_{d}^{\prime }$ are the hypotheses that “individual B is the offspring of an outbred female (individual A) with her outbred father (i.e., $\Delta =\left\{0,0,0,0,0.25,0,0.25,0.5,0\right\}$ between the mother–offspring pair)” and “individual B is the offspring of an outbred female (individual A) with a random outbred male (i.e., $\kappa =\left\{0,1,0\right\}$ between the mother–offspring pair)”. Thus, it can be unified as follows (see details in Section 2.1 of File S1 in Supplementary Materials):

$$II=\frac{d_{Ac}d_{Ad}}{2d_{Ac}p_{d}+2d_{Ad}p_{c}}+\frac{1}{2}$$ (19)

Furthermore, Eq. 19 can be generalized to a more comprehensive scenario, in which *H*_*p*_ represents the hypothesis “individual B is the offspring of two outbred relatives with $\kappa =\left\{\kappa_{0},\kappa_{1},\kappa_{2}\right\}$ , only one of which participated as individual A”. If we define $\left.\varphi =\kappa_{2}/2+\kappa_{1}/4\right.$ as the kinship coefficient (Brustad et al., 2021b) between the two alleged parents, it can be inferred that $\Delta =\left\{0,0,0,0,\varphi ,0,\varphi ,1-2\varphi ,0\right\}$ between individual A and B under this circumstance (see Section 2.2 of File S1 Supplementary Materials). Therefore, a more comprehensive form of *II* (*II*_*φ*_) can be computed as follows

$${II}_{\varphi }=\frac{2\varphi d_{Ac}d_{Ad}}{d_{Ac}p_{d}+d_{Ad}p_{c}}+\left(1-2\varphi \right)$$ (20)

### 2.4 The extension of the aforementioned calculation method to kinship identification involving multiple participants

#### 2.4.1 The basic method

The method of LR derivation in this work can be extended to identification involving multiple individuals. In such cases, LR can be calculated by considering the probability of a specific individual being a specific genotype from the perspective of IBD alleles of other individuals, and the probability of the genotype being the actual genotype detected. If this individual is labeled as individual B (with actual genotype of *cd*) and the others individuals R, then

$$LR=\frac{\mathrm{Pr}\left(R\left|H_{p}\right.\right)\times \mathrm{Pr}\left(B\left|R,H_{p}\right.\right)}{\mathrm{Pr}\left(R\left|H_{d}\right.\right)\times \mathrm{Pr}\left(B\left|R,H_{d}\right.\right)}$$ (21)

The latter probabilities in both Numerator and Denominator can be calculated as follows:

$$\begin{array}{cc} \mathrm{Pr}\left(B\left|R,H\right.\right) & =\underset{j}{\sum }\left[\mathrm{Pr}\left(B=G_{j}\left|R,H\right.\right)\times \mathrm{Pr}\left(B\equiv cd|B\equiv G_{j},R,H\right)\right]\\ & =\underset{j}{\sum }\left[\mathrm{Pr}\left(B\equiv G_{j}\left|R,H\right.\right)\times \mathrm{Pr}\left(G_{j}\equiv cd\right)\right] \end{array}$$ (22)

The second step in this equation is derived based on the same logic in section 2.3.1 when deriving Eq. 10, and probability $\mathrm{Pr}\left(G_{j}\equiv cd\right)$ can be calculated with Eq. 11. Moreover, similar to pairwise cases, the calculation results remain unchanged regardless whether ***c*** and ***d*** are identical, due to the constant factor canceling from the numerator and denominator.

#### 2.4.2 An example: “Standard” non-inbred trio cases

Consider the following non-inbred situation: 3 individuals participated the identification: a child (labeled as C, with detected genotype *cd*), one of his/her biological parents whose parentage has been confirmed (labeled as TP, with detected genotype *ab*), and an individual (labeled as AR, with detected genotype *ef*) who is unrelated to TP and alleged to be related to C under *H*_*p*_. LR can be calculated by taking the null hypothesis as *H*_*d*_, i.e., AR is unrelated to both TP and C. For such identification, C can be regarded as individual B mentioned in Eq. 21, and the other two participants as R, i.e.,

$$LR=\frac{\mathrm{Pr}\left(TP\equiv ab,AR\equiv ef\left|H_{p}\right.\right)}{\mathrm{Pr}\left(TP\equiv ab,AR\equiv ef\left|H_{d}\right.\right)}\times \frac{\mathrm{Pr}\left(C\equiv cd\left|TP\equiv ab,AR\equiv ef,H_{p}\right.\right)}{\mathrm{Pr}\left(C\equiv cd\left|TP\equiv ab,AR\equiv ef,H_{d}\right.\right)}$$ (23)

The relationship between TP and AR, which is unrelated in both hypotheses, remains constant. Therefore, LR equals the latter ratio in the above equation. The two probabilities in that ratio can still be calculated from the perspective of IBD alleles. If AR is unrelated to TP, the allele C inherited from TP must not be IBD to any of AR’s alleles, i.e., *κ*_2_ = 0 between C and AR. Thus their relationship under *H*_*p*_ can be described with *κ*_1_ between them, for example, *κ*_1_ = 1 if AR is alleged to be the other parent of C. If there is no other information, there can be 3 types of IBD allele C inherited from the other parent, *e*_*I*_, *f*_*I*_, and *x*_*I*_, with probabilities of $\left.\kappa_{1}/2\right.$ , $\left.\kappa_{1}/2\right.$ , and 1 − *κ*_1_, respectively. Considering that TP must pass *a*_*I*_ or *b*_*I*_ to C with equal probabilities, there can be 6 IBD genotypes of C under *H*_*p*_: *a*_*I*_ *e*_*I*_, *a*_*I*_ *f*_*I*_, *b*_*I*_ *e*_*I*_, *b*_*I*_ *f*_*I*_, each with a probability of $\left.\kappa_{1}/4\right.$ , as well as *a*_*I*_ *x*_*I*_ and *b*_*I*_ *x*_*I*_, each with a probability of 1/2 − *κ*_1_/2; Meanwhile, there can be 2 type of C’s IBD genotype under *H*_*d*_: *a*_*I*_ *x*_*I*_ and *b*_*I*_ *x*_*I*_, each with a probability of 1/2. Thus, according to Eq. 22, LR can be calculated as follows:

$$LR=\frac{\frac{\kappa_{1}}{4}\left[\mathrm{Pr}\left(a_{I}e_{I}\equiv cd\right)+\mathrm{Pr}\left(a_{I}f_{I}\equiv cd\right)+\mathrm{Pr}\left(b_{I}e_{I}\equiv cd\right)+\mathrm{Pr}\left(b_{I}f_{I}\equiv cd\right)\right]}{\frac{1}{2}\left[\mathrm{Pr}\left(a_{I}x_{I}\equiv cd\right)+\mathrm{Pr}\left(b_{I}x_{I}\equiv cd\right)\right]}+\left(1-\kappa_{1}\right)$$ (24)

Each probability in the equation can be calculated according to Eq. 11 considering the fact that no IBD relationship should exist among the four alleles of TP and AR, if the non-inbred assumption is accepted. In summary, LR in non-inbred trio cases can be calculated as follows:

$$LR=\frac{\kappa_{1}d_{\mathrm{TPc}}d_{\mathrm{ARd}}+\kappa_{1}d_{\mathrm{TPd}}d_{\mathrm{ARc}}}{2d_{\mathrm{TPc}}p_{d}+2d_{\mathrm{TPd}}p_{c}}+\left(1-\kappa_{1}\right)$$ (25)

Two more examples of LR calculation in identifications involving multiple participants are given in Sections 4.3.7 and 4.3.8 of File S1 in Supplementary Materials.

### 2.5 Verification of the unification results

#### 2.5.1 For inbred identification

The 1 × 9 results of $\left.\mathrm{Pr}\left(E\left|J_{i}\right.\right)/\mathrm{Pr}\left(E\left|J_{9}\right.\right)\right.$ unification can be verified by comparison with Table 1 of Brustad et al. (2021b) (which is in 9 × 9 form) under the nine possible genotype combination types of the participants. The simplest situation, in which both individual A and B are homozygous, *ii*, is given as example in Table 1; as shown in the table, the results obtained with the two methods were identical under every Jacquard state. This identity persisted for the other eight genotype combinations, as shown in Section 3 of File S1 in Supplementary Materials.

**TABLE 1:** Example of verification of $\mathrm{Pr}\left(E|J_{i}\right)/\mathrm{Pr}\left(E|J_{9}\right)$ unification.

		Results				Results	
*J*_*i*_	Unified ratio	Unified^a^	Listed^b^	*J*_*i*_	Unified ratio	Unified^a^	Listed^b^
*J*_1_	$\frac{1_{ab}1_{ac}1_{cd}}{p_{a}^{3}}$	$\frac{1\times 1\times 1}{p_{a}^{3}}=\frac{1}{p_{i}^{3}}$	$\frac{p_{i}}{p_{i}^{4}}$	*J*_2_	$\frac{1_{ab}1_{cd}}{p_{a}p_{c}}$	$\frac{1\times 1}{p_{i}\times p_{i}}=\frac{1}{p_{i}^{2}}$	$\frac{p_{i}^{2}}{p_{i}^{4}}$
*J*_3_	$\frac{1_{ab}\left(1_{ac}+1_{ad}\right)}{2p_{a}^{2}}$	$\frac{1\times \left(1+1\right)}{2\times p_{i}^{2}}=\frac{1}{p_{i}^{2}}$	$\frac{p_{i}^{2}}{p_{i}^{4}}$	*J*_4_	$\frac{1_{ab}}{p_{a}}$	$\frac{1}{p_{i}}$	$\frac{p_{i}^{3}}{p_{i}^{4}}$
*J*_5_	$\frac{1_{cd}\left(1_{ac}+1_{bc}\right)}{2p_{c}^{2}}$	$\frac{1\times \left(1+1\right)}{2\times p_{i}^{2}}=\frac{1}{p_{i}^{2}}$	$\frac{p_{i}^{2}}{p_{i}^{4}}$	*J*_6_	$\frac{1_{cd}}{p_{c}}$	$\frac{1}{p_{i}}$	$\frac{p_{i}^{3}}{p_{i}^{4}}$
*J*_7_	$\frac{1_{ac}1_{bd}+1_{ad}1_{bc}}{2p_{c}p_{d}}$	$\frac{1\times 1+1\times 1}{2\times p_{i}\times p_{i}}=\frac{1}{p_{i}^{2}}$	$\frac{p_{i}^{2}}{p_{i}^{4}}$	*J*_8_	$\frac{d_{c}p_{d}+d_{d}p_{c}}{4p_{c}p_{d}}$	$\frac{2\times p_{i}+2\times p_{i}}{4\times p_{i}\times p_{i}}=\frac{1}{p_{i}}$	$\frac{p_{i}^{3}}{p_{i}^{4}}$
*J*_9_	1	1	$\frac{p_{i}^{4}}{p_{i}^{4}}$				

a Notes:^*^ $1_{ab}=1_{ac}=1_{ad}=1_{bc}=1_{bd}=1_{cd}=1$ , *d*_*c*_ = *d*_*d*_ = 2 and *p*_*a*_ = *p*_*c*_ = *p*_*d*_ = *p*_*i*_ when both individual A and B are homozygotes *ii*.

^b^ Results calculated according to the first row of Table 1 in Brustad et al. (2021b), in which the alleles of the two individuals were “*a*” and have been adjusted to “*i*” to avoid confusion with the parameters in the unified formulae.

#### 2.5.2 For non-inbred identification

We reproduce the unified formula derived by (formula (2.19) in Egeland et al. (2017)) as Eq. 26 in this paper. If mutation is not considered, $m_{ij}^{\left(n\right)}=1_{ij}$ , where $m_{ij}^{\left(n\right)}$ represents the probability that allele *i* becomes *j* after *n* cycles of meiosis. Thus, Eq. 16 is equivalent to Eq. 26 when we do not consider mutation.

$$LR=\kappa_{0}+\kappa_{1}\frac{\left(m_{ac}^{\left(n\right)}+m_{bc}^{\left(n\right)}\right)p_{d}+\left(m_{ad}^{\left(n\right)}+m_{bd}^{\left(n\right)}\right)p_{c}}{4p_{c}p_{d}}+\kappa_{2}\frac{m_{ac}^{\left(n\right)}m_{bd}^{\left(n\right)}+m_{ad}^{\left(n\right)}m_{bc}^{\left(n\right)}}{2p_{c}p_{d}}$$ (26)

#### 2.5.3 For alleged father-daughter incest cases

II was calculated and listed according to different genotype combination of the two individuals in Table 1 of Wenk (2007). The comparison of results calculated by Eq. 19 with these listed results is provided in Table 2, showing that the two methods are equivalent to each other under every possible combination type of the mother-offspring pair.

**TABLE 2:** Comparison between the unified formula with listed ones in the calculation of incest index.

Genotype			Unified calculation as Eq. 19				
Mother	Child	Listed result derived by Wenk	*d*_*c*_	*d*_*d*_	*p*_*c*_	*p*_*d*_	Result
A/A	A/A	$\left(0.5a+0.5\right)/a$	2	2	*a*	*a*	1/2 + 1/2*a*
	A/B	0.5	2	0	*a*	*b*	1/2
A/B	A/A	$\left(0.5a+0.25\right)/a$	1	1	*a*	*a*	1/2 + 1/4*a*
	A/C	0.5	1	0	*a*	*c*	1/2
	A/B	$\left(0.5a+0.5b+0.5\right)/\left(a+b\right)$	1	1	*a*	*b*	$1/2+1/\left(2a+2b\right)$

Note: Symbols here are adjusted in the form used by Table 1 of Wenk (2007): alleles in the genotypes of the two participants are represented with uppercase letters “A”, “B” and “C”, which are different to each other; The corresponding lowercase letters “*a*”, “*b*” and “*c*” denote the frequency of these alleles in the population.

#### 2.5.4 For trio paternity testing

“Standard” trio paternity testing is a routine procedure in forensic genetic practice, involving the evaluation of the paternity of a male (AF) to a child (C) with the genetic information of C’s mother (M). This testing can be considered as a specific scenario of standard non-inbred trio cases discussed in section 2.4.2, where AR is AF, TP is M, and *κ*_1_ = 1. Consequently, the paternity index in these cases, denoted as *PI*_*trio*_, can be calculated as follows:

$${PI}_{\mathrm{trio}}=\frac{d_{Mc}d_{\mathrm{AFd}}+d_{Md}d_{\mathrm{AFc}}}{2d_{Mc}p_{d}+2d_{Md}p_{c}}$$ (27)

The comparison of results calculated by Eq. 27 with those calculated by the recognized list formed methods (Fung and Hu, 2007) is listed in Table 3, showing that the two methods are equivalent to each other under every possible combination type of the three participants.

**TABLE 3:** Comparison between the unified LR formula with listed ones in standard trio paternity testing.

Genotype			Listed result derived by Fung *et al*	Unified calculation as Eq. 27						
C	M	AF		*d*_*Mc*_	*d*_*Md*_	*d*_*AFc*_	*d*_*AFd*_	*p*_*c*_	*p*_*d*_	Result
*A*_*i*_ *A*_*i*_	*A*_*i*_ *A*_*i*_	*A*_*i*_ *A*_*i*_	$\left.1/p_{i}\right.$	2	2	2	2	*p*_*i*_	*p*_*i*_	$\left.8/\left(4p_{i}+4p_{i}\right)=1/p_{i}\right.$
		*A*_*i*_ *A*_*j*_	$\left.1/\left(2p_{i}\right)\right.$	2	2	1	1	*p*_*i*_	*p*_*i*_	$\left.4/\left(4p_{i}+4p_{i}\right)=1/\left(2p_{i}\right)\right.$
	*A*_*i*_ *A*_*j*_	*A*_*i*_ *A*_*i*_	$\left.1/p_{i}\right.$	1	1	2	2	*p*_*i*_	*p*_*i*_	$\left.4/\left(2p_{i}+2p_{i}\right)=1/p_{i}\right.$
		*A*_*i*_ *A*_*j*_	$\left.1/\left(2p_{i}\right)\right.$	1	1	1	1	*p*_*i*_	*p*_*i*_	$\left.2/\left(2p_{i}+2p_{i}\right)=1/\left(2p_{i}\right)\right.$
		*A*_*i*_ *A*_*k*_	$\left.1/\left(2p_{i}\right)\right.$	1	1	1	1	*p*_*i*_	*p*_*i*_	$\left.2/\left(2p_{i}+2p_{i}\right)=1/\left(2p_{i}\right)\right.$
*A*_*i*_ *A*_*j*_	*A*_*i*_ *A*_*i*_	*A*_*i*_ *A*_*j*_	$\left.1/\left(2p_{j}\right)\right.$	2	0	1	1	*p*_*i*_	*p*_*j*_	$\left.2/\left(0p_{i}+4p_{j}\right)=1/\left(2p_{j}\right)\right.$
		*A*_*j*_ *A*_*j*_	$\left.1/p_{j}\right.$	2	0	0	2	*p*_*i*_	*p*_*j*_	$\left.4/\left(0p_{i}+4p_{j}\right)=1/p_{j}\right.$
		*A*_*j*_ *A*_*k*_	$\left.1/\left(2p_{j}\right)\right.$	2	0	0	1	*p*_*i*_	*p*_*j*_	$\left.2/\left(0p_{i}+4p_{j}\right)=1/\left(2p_{j}\right)\right.$
	*A*_*i*_ *A*_*j*_	*A*_*i*_ *A*_*i*_	$\left.1/\left(p_{i}+p_{j}\right)\right.$	1	1	2	0	*p*_*i*_	*p*_*j*_	$\left.2/\left(2p_{i}+2p_{j}\right)=1/\left(p_{i}+p_{j}\right)\right.$
		*A*_*i*_ *A*_*j*_	$\left.1/\left(p_{i}+p_{j}\right)\right.$	1	1	1	1	*p*_*i*_	*p*_*j*_	$\left.2/\left(2p_{i}+2p_{j}\right)=1/\left(p_{i}+p_{j}\right)\right.$
		*A*_*i*_ *A*_*k*_	$\left.1/\left[2\left(p_{i}+p_{j}\right)\right]\right.$	1	1	1	0	*p*_*i*_	*p*_*j*_	$\left.1/\left(2p_{i}+2p_{j}\right)=1/\left[2\left(p_{i}+p_{j}\right)\right]\right.$
	*A*_*i*_ *A*_*k*_	*A*_*i*_ *A*_*j*_	$\left.1/\left(2p_{j}\right)\right.$	1	0	1	1	*p*_*i*_	*p*_*j*_	$\left.1/\left(0p_{i}+2p_{j}\right)=1/\left(2p_{j}\right)\right.$
		*A*_*j*_ *A*_*j*_	$\left.1/p_{j}\right.$	1	0	0	2	*p*_*i*_	*p*_*j*_	$\left.2/\left(0p_{i}+2p_{j}\right)=1/p_{j}\right.$
		*A*_*j*_ *A*_*k*_	$\left.1/\left(2p_{j}\right)\right.$	1	0	0	1	*p*_*i*_	*p*_*j*_	$\left.1/\left(0p_{i}+2p_{j}\right)=1/\left(2p_{j}\right)\right.$
		*A*_*j*_ *A*_*l*_	$\left.1/\left(2p_{j}\right)\right.$	1	0	0	1	*p*_*i*_	*p*_*j*_	$\left.1/\left(0p_{i}+2p_{j}\right)=1/\left(2p_{j}\right)\right.$

Note: Symbols here are adjusted in the form used by Table 4.1 of Fung and Hu (2007): alleles in the genotypes of the three participants are represented with “***A***_**…**_ ” and the corresponding frequencies in the population are denoted with “***p***_**…**_ “, where different subscripts means different alleles.

### 2.6 *KINSIMU*: A new series of simulation tools to promote research in kinship analysis

In order to evaluate the efficacy of specific panels in kinship identification, simulation can be a useful tool. Based on the unified formulae of LR calculation in pairwise kinship analysis, a series of newly defined functions is constructed for kinship simulation and calculation on the R (4.2.1) platform, an archive file is provided in File S2 in Supplementary Materials. Simulation can be divided into two steps: i) generation of genotype data for a specific number of participants (i.e., the sample size, labeled as “ss”) with specific relationships; and ii) calculation of specific parameters for each case, e.g., identity by state score (IBS), or different type of LR. If independence among all the markers is assumed, the simulations at each locus should not affect each other, and the calculation results at each locus can be directly accumulated or multiplied. Thus, the whole code can be written in a one-layer loop, as shown in Algorithm 1. In each loop, the genotype data are replaced and the IBS/log_10_LR results are accumulated with the previous calculation. The above process can be completed using function “*testsimulation* ()”. Detailed instructions for use of the functions is given in Section 4 of File S1 in Supplementary Materials.

Algorithm 1 Typical process of simulation with *KINSIMU* package.

**Input:** The allele frequency data
**Output:** A data frame containing calculation results
1 Input the allele frequency by hand or import the frequency data with function “*EvaluatPanel*()”;
2 Extract the number of loci (“*nl*”)
3 Set the sample size (“*ss*”), the true relationship between/among the individuals (“*tdelta*”), and the alleged relationship in LR calculation (“*adelta*”);
4 Create data frame containing the results with initial values of 0;
5 **for** *i = 1:nl* **do**
6  Simulate genotype combination of *ss* pairs/groups of individual cosidering *tdelta* with function “*pairsimu*()” or “*pedisimu*()”, based on the frequency data of *i*th marker;
7  Calculate LR or identity by state (IBS) score for each pair/group with function “*LRparas* ()”, “*IICAL*()”, “*TrioPI*()”, *etc.*, based on unified equations. Note that the base 10 logarithms of LRs calculated will be output;
8  Accumulate output results to the result data frame;
9 **end**

#### 2.6.1 Evaluation of the package

Multiple types of evaluation were performed on the function “*testsimulation*()” based on allele frequency data for 42 autosomal short tandem repeat (STR) markers (Liu et al., 2020) in a Chinese Han population, given as data “*FortytwoSTR*” in the package. Similar simulations were carried out using two existing tools, *Familias* 3.3 (Egeland et al., 2016) and R package *relSim* (Curran, 2023), for comparison. All simulations were performed on the same personal device with Intel^®^ Core™ i7-9700F CPU and 16 GB RAM, and the code used in the evaluation is given in File S3 in Supplementary Materials.

In essence, the main purpose of simulation (as well as the integration of LR formulae) is not only to compute precise results for individual real-life cases, but also to reveal the overall pattern of specific parameters within a particular type of identification. Therefore, in addition to the accuracy of the calculation results for a single case, the stability of result distribution in a large sample size is also crucial for the simulation package. Given that we have previously proven exhaustively in section 2.5 that the unified formula produces consistent results with the list format for specific single cases, our attention in this comparative analysis is primarily directed towards the following aspects: i) Identify any disparities in the distribution of calculated results between *KINSIMU* with the two existing tools, after a significant number of simulations. In other words, analyze whether there is a bias towards simulating specific genotype combinations with *KINSIMU*; ii) Assess the speed of simulation execution. As shown in Section 5 of File S1 in Supplementary Materials, the package *KINSIMU* can stably simulate and calculate multiple kinship cases with a speed of about 6,000,000 loci per second (as shown in Figure S4 of File S1 in Supplementary Materials). When simulating individual pairs with the same relationships and calculating the same parameters, the running speed of the *KINSIMU* package is approximately 10 times that of *relSim* (see Figure S3D of File S1 in Supplementary Materials) and at least 80 times that of *Familias* 3.3 (see Section 5.2 of File S1 in Supplementary Materials).

#### 2.6.2 Application of the package

The above tools have been used in the construction of next-generation sequencing kits containing multiple single-nucleotide polymorphism (Zhao et al., 2021) or microhaplotype (Du et al., 2023) markers. In the relevant studies, family surveys were carried out at the same time, and the results of the two types of research were found to be similar; see Fig. 4 of (Zhao et al., 2021) and Fig. 4 of (Du et al. 2023), which illustrate the effectiveness of the simulation tools in real-life cases.

## 3 Discussion

In this work, a new approach in the unification of LR formulae has been introduced, the core of which is based on the perspective of IBD alleles and IBD genotypes. Based on the formulae, a package named *KINSIMU* is constructed for large-sample-size simulation research. Although some prototype software is already available for pedigree simulations (such as the two methods we compared with *KINSIMU*), any such tool (including *KINSIMU*) has limitations and may not take every possibility into account. Therefore, it may be more convenient for researchers to write their own simulation and calculation code for specific complex situations. In such cases, the coding logic and concepts of existing tools can be used for reference. The aim of this paper was to develop unified formulae for LR calculation and to simplify the coding process. Based on these formulae, we are making our tools for kinship simulation open-source and suggest that other researchers do the same to help the development of the discipline.

In simulation studies involving LR methods, the commonly used list-form presentation tends to obstruct the self-coding process. By using the most simplified formulae provided by the list-form method, the LR calculation process would involve two multiple-to-one choosing tasks: i) determining the type of the participants’ genotype combination at a specific locus from multiple possible ones; and ii) judging the position of each allele in the specific combination. These tasks would vary at different loci, requiring locus by locus or type by type calculations for LR, necessitating logical comparison functions like “if()”, leading to increased coding complexity. Additionally, all possible participant combinations must be considered in the coding process, or errors may occur in certain cases. In contrast, no logical comparison function would be required when using unified formulae; based on the genotype data, nine or six parameters (depending on whether inbreeding factors are considered) could be uniformly calculated and then brought into a unified formula for identification.

In most studies that utilize *Familias* (Gonçalves et al., 2017; Li et al., 2019; Wu et al., 2021; Pilli et al., 2022), the sample size for simulations is often less than 10,000 due to the substantial increase in memory requirements when simulating larger samples. This limitation may be attributed to the tool’s operation method, wherein genotype data is retained until the end of the simulation, leading to unnecessary memory occupation. Contrarily, R packages like *KINSIMU* or *relSim* replace the data per loop (or per “Block”) after it is used for calculation. It is somewhat “unfair” to compare the running time of our R tools with the stand-alone software *Familias* instead of its R version (which is no longer directly accessible from CRAN, and the available installation package is not compatible with our R version). However, some insights can be gained from this comparison. For instance, with *Familias*, the more complex the relationship between participants, the longer the running time, even with the same sample size. This phenomenon is not observed with *KINSIMU*, possibly because we directly generate the participants’ genotypes rather than determining them through parent-child inheritance, resulting in a significant reduction in the needed random numbers during simulation. However, the parent-child inheritance approach may be necessary in cases with complicated pedigrees or when more individuals need to be considered for calculating LR. Therefore, we provide an approach for such cases in the form of the function “*pedisimu*()”. Meanwhile, it is important to note that the tools *Familias* and *relSim* offer numerous functions besides simulation.

The direct-generation approach is used in the package *relSim*, and the simulation process is divided into 100 sub-fractions (so-called “Blocks”), each of which is replaced by the next. As a result, it can simulate at least 10,000,000 pairs in a single process on our device. When the sample size is no longer an obstacle, the running time becomes the main bottleneck in large-sample simulation research. In the reference manual of *relSim* package, the authors state that it would take at least 30 h on a personal device to perform a simulation of 300,000,000 individual pairs on 13 CODIS loci (Balding et al., 2013). Even allowing for decreased running time with ongoing updates to devices, the latest version of *relSim* would still take twice as long as *KINSIMU* to carry out the same simulation. Examination of the functions of *relSim* shows that genotype data are cached per case but generated per locus. Therefore, an extra “for” loop layer is required to allocate the data. Another extra loop layer exists for the calculation of CIBS or CLR with *relSim*, which would take place per case per locus when applying list-form formulae. Furthermore, at least two layers of “if (…)” functions are needed in these calculations to classify the genotype combinations in different situations and to calculate the parameters with different equations. It is difficult to apply such methods to multiple cases per locus, as can be done with the unified functions in *KINSIMU*; this may be the main cause of the difference in running time between the two packages. A side-by-side comparison of the codes used in PI calculation is given in Section 6 of File S1 in Supplementary Materials.

The method introduced in this paper for LR unification in kinship testing can be regarded as a divide-and-conquer method. More specifically, the problem is essentially about the genotype inference of individual B from the perspective of IBD under different hypotheses. This problem can be solved with the following approach: i) division of the original problem into several independent easy-to-solve sub-problems, i.e., the division of different IBD states; ii) conquering each sub-problem, i.e., calculating Δ/*κ* and the ratio of conditional probabilities under each state; and iii) combining the solutions to the sub-problems into the solution for the original problem. The use of sub-scripted letters (e.g., *a*_*I*_) for individual B’s genotype from the perspective of IBD alleles eliminates the differences in the detected genotype combinations of the two individuals. Therefore, the derivation in step ii of each sub-problem can be done in a unified way; this is simpler in some ways than the method Egeland *et al*. Introduced in Appendix A of (Egeland et al., 2017), in which the combinations had to be listed exhaustively. Thus, the derivation can be easily extended to identifications considering inbreeding factors or involving multiple individuals.

Mutation is not considered in the inference process in the present work, resulting in underestimation of the LR value in some cases. However, we argue that the impact of mutation on the LR value is relatively small in identifications where LR cannot be 0. For example, in pairwise identification using STR markers, as discussed in (Egeland et al., 2017), the probability of mutation occurring is relatively low in parentage identification and increases with the number of cycles of meiosis. In other words, the probability is larger when the relationship between the two individuals is more distant. In that case, *κ*_0_ would be larger, and the other two *κ* parameters (i.e., the coefficients of parts in the LR formula related to the mutation) would be smaller, which limits the impact of mutation on the LR value to a relatively low level. Furthermore, the mutation factor can be introduced into the calculation if 1 parameters are replaced by corresponding *m* ones.

This is a preliminary study on the concept of unification of LR calculation in kinship identification; further work based on our findings could include the inference of LR in cases involving multiple individuals or where linked markers are available.

## Data Availability

The original contributions presented in the study are included in the article/Supplementary Material, further inquiries can be directed to the corresponding author.
