# Supplementary material for: An approach to unified formulae for likelihood ratio calculation in pairwise kinship analysis
Source: Front Genet. 2024 Feb 7;15:1226228. doi: 10.3389/fgene.2024.1226228 (PMC10879572; doi:10.3389/fgene.2024.1226228)
Supplement: Supplementary file 1 [file DataSheet1.ZIP › File_S1.pdf]

Supplementary material of "An approach to unified formulae for likelihood ratio calculation in pairwise kinship analysis"

Contents

1 Unification of ratio $\Pr(E|J_i)/\Pr(E|J_9)$ 2

2 Detailed derivation related to incest index (II) 3
2.1 II in father-daughter incest cases . . . 3
2.2 II when the alleged parents are related with $\kappa = \{\kappa_0, \kappa_1, \kappa_2\}$ . . . 3
2.2.1 Derivation of the Δ distribution under H_p . . . 3
2.2.2 Derivation of II_φ . . . 3

3 Verification of the unification results 4

4 Instruction of functions of R package *KINSIMU* 5
4.1 Brief introduction . . . 5
4.2 Install and library the *KINSIMU* package . . . 5
4.3 Functions in the package . . . 5
4.3.1 '*EvaluatePanel*()' . . . 5
4.3.2 '*pairsimu*()' . . . 7
4.3.3 '*pedisimu*()' . . . 9
4.3.4 '*LRparas*()' . . . 10
4.3.5 '*trioPI*()' . . . 12
4.3.6 '*IICAL*()' . . . 13
4.3.7 '*LRhsip*()' . . . 14
4.3.8 '*LRgpgcam*()' . . . 16
4.3.9 '*logLR*()' . . . 18
4.3.10 '*testsimulation*()' . . . 19
4.3.11 '*outputCSV*()' . . . 20
4.4 Data involved . . . 20
4.4.1 *FortytwoSTR* . . . 20
4.4.2 *pediexample* . . . 21

5 Evaluation of R package *KINSIMU* 22
5.1 Stability . . . 22
5.2 Inter-platform similarity . . . 23
5.3 Inner-platform similarity . . . 24
5.4 Running speed estimation . . . 24

6 Comparison between the PI calculation codes of *KINSIMU* and *relSim* 25
6.1 *KINSIMU* code . . . 25
6.2 *relSim* code . . . 25

Symbols in this Supplement file

Symbols in this supplemental file are defined in the same manner as in the main text. To facilitate readers' comprehension, we present an additional summary and introduction of them: **(i)** ***E***: The detected DNA evidence; **(ii)** J_i: The 9 Jacquard states; **(iii)** $\mathcal{A}_{j1}$ and $\mathcal{A}_{j2}$: The two IBD alleles of individual B under specific situation; **(iv)** "≡": Specific genotypes or alleles are identical to each other; **(v)** ***a, b, c*** and ***d***: Detected alleles of specific individuals, which can be identical to each other or not; **(vi)** a_I and b_I: The IBD allele of the corresponding allele; **(vii)** x_I and y_I: random alleles not IBD to a or b; **(viii)** "$p_{...}$": Frequency of the corresponding allele; **(ix)** "$d_{A...}$": The dosage of the corresponding allele in the genotype of individual A (note that only deduction related to pairwise situation is discussed in this supplemental file); **(x)** "$\mathbb{1}_{...}$: Conditional parameter which equals 1 if alleles represented by the two subscript letters are identical and 0 otherwise.

1 Unification of ratio $\Pr(E|J_i)/\Pr(E|J_9)$

The process unifying the ratio ratio $\Pr(E|J_i)/\Pr(E|J_9)$, is presented in Table S1 of this file.

Table S1: Unification of ratio $\Pr(E|J_i)/\Pr(E|J_9)$

J_i	$\frac{\Pr(A\equiv ab\|J_i)}{\Pr(A\equiv ab\|J_9)}$	$\mathcal{G}_j$				$\Pr(\mathcal{G}_j\equiv cd)$	$\frac{\Pr(B\equiv cd\|A\equiv ab,J_i)}{\Pr(B\equiv cd\|A\equiv ab,J_9)}$ †	$\frac{\Pr(E\|J_i)}{\Pr(E\|J_9)}$ ‡
		j	$\mathcal{A}_{j1}$	$\mathcal{A}_{j2}$	$\Pr_1$*			
J_1	$\frac{\mathbb{1}_{ab}}{p_a}$	1	a_I	a_I	1	$2\mathbb{1}_{ac}\mathbb{1}_{cd}$	$\frac{\mathbb{1}_{ac}\mathbb{1}_{cd}}{p_a^2}$ #	$\frac{\mathbb{1}_{ab}\mathbb{1}_{ac}\mathbb{1}_{cd}}{p_a^3}$
J_2	$\frac{\mathbb{1}_{ab}}{p_a}$	1	x_I	x_I	1	$2p_c\mathbb{1}_{cd}$	$\frac{\mathbb{1}_{cd}}{p_c}$	$\frac{\mathbb{1}_{ab}\mathbb{1}_{cd}}{p_ap_c}$
J_3	$\frac{\mathbb{1}_{ab}}{p_a}$	1	a_I	x_I	1/2	$p_d\mathbb{1}_{ac}+p_c\mathbb{1}_{ad}$	$\frac{\mathbb{1}_{ac}+\mathbb{1}_{ad}}{2p_a}$	$\frac{\mathbb{1}_{ab}(\mathbb{1}_{ac}+\mathbb{1}_{ad})}{2p_a^2}$
			x_I	a_I	1/2	$p_c\mathbb{1}_{ad}+p_d\mathbb{1}_{ac}$		
J_4	$\frac{\mathbb{1}_{ab}}{p_a}$	1	x_I	y_I	1	$2p_cp_d$	1	$\frac{\mathbb{1}_{ab}}{p_a}$
J_5	1	1	a_I	a_I	1/2	$2\mathbb{1}_{ac}\mathbb{1}_{cd}$	$\frac{\mathbb{1}_{ac}\mathbb{1}_{cd}+\mathbb{1}_{bc}\mathbb{1}_{cd}}{2p_c^2}$	$\frac{\mathbb{1}_{cd}(\mathbb{1}_{ac}+\mathbb{1}_{bc})}{2p_c^2}$
		2	b_I	b_I	1/2	$2\mathbb{1}_{bc}\mathbb{1}_{cd}$		
J_6	1	1	x_I	x_I	1	$2p_c\mathbb{1}_{cd}$	$\frac{\mathbb{1}_{cd}}{p_c}$	$\frac{\mathbb{1}_{cd}}{p_c}$
J_7	1	1	a_I	b_I	1/2	$\mathbb{1}_{ac}\mathbb{1}_{bd}+\mathbb{1}_{ad}\mathbb{1}_{bc}$	$\frac{\mathbb{1}_{ac}\mathbb{1}_{bd}+\mathbb{1}_{ad}\mathbb{1}_{bc}}{2p_cp_d}$	$\frac{\mathbb{1}_{ac}\mathbb{1}_{bd}+\mathbb{1}_{ad}\mathbb{1}_{bc}}{2p_cp_d}$
		2	b_I	a_I	1/2	$\mathbb{1}_{bc}\mathbb{1}_{ad}+\mathbb{1}_{bd}\mathbb{1}_{ac}$		
J_8	1	1	a_I	x_I	1/4	$p_d\mathbb{1}_{ac}+p_c\mathbb{1}_{ad}$	$\frac{p_cd_{Ad}+p_dd_{Ac}}{4p_cp_d}$	$\frac{p_cd_{Ad}+p_dd_{Ac}}{4p_cp_d}$
		2	x_I	a_I	1/4	$p_c\mathbb{1}_{ad}+p_d\mathbb{1}_{ac}$		
		3	b_I	x_I	1/4	$p_d\mathbb{1}_{bc}+p_c\mathbb{1}_{bd}$		
		4	x_I	b_I	1/4	$p_c\mathbb{1}_{bd}+p_d\mathbb{1}_{bc}$		
J_9	1	1	x_I	y_I	1	$2p_cp_d$	1	1

* $\Pr_1 = \Pr\left(B\equiv\mathcal{G}_j\middle|A\equiv ab,J_i\right)$;

† $\Pr(B\equiv cd|A\equiv ab,J_i)=\sum_j\left[\Pr\left(B\equiv\mathcal{G}_j\middle|A\equiv ab,J_i\right)\times\Pr\left(\mathcal{G}_j\equiv cd\right)\right]$;

‡ $\frac{\Pr(E|J_i)}{\Pr(E|J_9)}=\frac{\Pr(A\equiv ab|J_i)}{\Pr(A\equiv ab|J_9)}\times\frac{\Pr(B\equiv cd|A\equiv ab,J_i)}{\Pr(B\equiv cd|A\equiv ab,J_9)}$

\# Ratios in this column are derived and transformed according the following facts: $\mathbb{1}_{ij}/p_i=\mathbb{1}_{ij}/p_j$;

2 Detailed derivation related to incest index (II)

2.1 II in father-daughter incest cases

As discussed in the main text, the incest index can be calculated as the ratio of two LRs with identical Hd:

$$II = \frac{LR(\Delta = \{0,0,0,0,0.25,0,0.25,0.5,0\})}{LR(\kappa = \{0,1,0\})} \tag{S1}$$

According to Eq. (15) in the main text, the formula can be further derived as follows:

$$II = \frac{\frac{\mathbb{1}_{cd}(\mathbb{1}_{ac}+\mathbb{1}_{bc})}{8p_c^2} + \frac{\mathbb{1}_{ac}\mathbb{1}_{bd}+\mathbb{1}_{ad}\mathbb{1}_{bc}}{8p_cp_d} + \frac{d_{Ac}p_d+d_{Ad}p_c}{8p_cp_d}}{\frac{d_{Ac}p_d+d_{Ad}p_c}{4p_cp_d}} \tag{S2}$$

Considering $\mathbb{1}_{cd}/p_c^2 = \mathbb{1}_{cd}/p_cp_d$ and $\mathbb{1}_{ij}\mathbb{1}_{jk} = \mathbb{1}_{ij}\mathbb{1}_{ik}$, it can be seen that

$$\begin{aligned} II &= \frac{\frac{\mathbb{1}_{cd}(\mathbb{1}_{ac}+\mathbb{1}_{bc})}{8p_cp_d} + \frac{\mathbb{1}_{ac}\mathbb{1}_{bd}+\mathbb{1}_{ad}\mathbb{1}_{bc}}{8p_cp_d} + \frac{d_{Ac}p_d+d_{Ad}p_c}{8p_cp_d}}{\frac{d_{Ac}p_d+d_{Ad}p_c}{4p_cp_d}} \\ &= \frac{\mathbb{1}_{ad}\mathbb{1}_{ac} + \mathbb{1}_{bd}\mathbb{1}_{bc} + \mathbb{1}_{ac}\mathbb{1}_{bd} + \mathbb{1}_{ad}\mathbb{1}_{bd}}{2d_{Ac}p_d + 2d_{Ad}p_c} + \frac{1}{2} \\ &= \frac{d_{Ac}d_{Ad}}{2d_{Ac}p_d + 2d_{Ad}p_c} + \frac{1}{2} \end{aligned} \tag{S3}$$

2.2 II when the alleged parents are related with $\kappa = \left\{\kappa_0, \kappa_1, \kappa_2\right\}$

2.2.1 Derivation of the Δ distribution under H_p

If individual A is assumed outbred, $J_1 \sim J_4$ cannot happen. Meanwhile, if individual B is individual A's offspring, he/she must inherited an allele from individual A. Thus, J_6 and $J-9$ cannot happen. In other words, $\Delta_1 = \Delta_2 = \Delta_3 = \Delta_4 = \Delta_6 = \Delta_9 = 0$.

If individual B's father is related with individual A with $\kappa = \{\kappa_0, \kappa_1, \kappa_2\}$, there are 4 possible types of his IBD genotypes: a_Ib_I, a_Ix_I, b_Ix_I, and x_Iy_I with probabilities of κ_2, $\kappa_1/2$, $\kappa_1/2$ and κ_0, respectively. Thus, there are 3 types of alleles he can pass to individual B from the perspective of IBD alleles: a_I, b_I and x_I with probabilities of φ, φ and $(1-2\varphi)$, respectively. Considering that individual A would pass a_I or b_I to individual B with equal probabilities, there are 5 types of individual B's IBD genotype: a_Ia_I and b_Ib_I belonging to J_5, with probabilities of $\varphi/2$, respectively; a_Ib_I belonging to J_7 with probability of φ; a_Ix_I and b_Ix_I belonging to J_8, with probabilities of $1/2 - \varphi$, respectively. Thus, $\Delta = \{0,0,0,0,\varphi,0,\varphi,1-2\varphi,0\}$ under H_p.

2.2.2 Derivation of II_φ

With the derivation similar to Eq. (S1), it can be seen that

$$\begin{aligned} II_\varphi &= \frac{LR(\Delta = \{0,0,0,0,\varphi,0,\varphi,1-2\varphi,0\})}{LR(\kappa = \{0,1,0\})} \\ &= \frac{\frac{\varphi(\mathbb{1}_{ac}\mathbb{1}_{ad}+\mathbb{1}_{bc}\mathbb{1}_{bd})}{4p_cp_d} + \frac{\varphi\mathbb{1}_{ac}\mathbb{1}_{bd}+\mathbb{1}_{ad}\mathbb{1}_{bc}}{4p_cp_d} + \frac{(1-2\varphi)(d_{Ac}p_d+d_{Ad}p_c)}{4p_cp_d}}{\frac{d_{Ac}p_d+d_{Ad}p_c}{4p_cp_d}} \\ &= \frac{\varphi d_{Ac}d_{Ad}}{2d_{Ac}p_d + 2d_{Ad}p_c} + (1-2\varphi) \end{aligned} \tag{S4}$$

3 Verification of the unification results

Verification of the unification results when the two participants have different genotype combinations (i.e., under the 8 situations other than that presented in the main text) is listed in Table S2∼S3 in this file: Table S2: **Situation ii)** $A = ii, B = jj$, **Situation iii)** $A = ii, B = ij$, **Situation iv)** $A = ii, B = jk$, and **Situation v)** $A = ij, B = ii$; Table S3: **Situation vi)** $A = jk, B = ii$, **Situation vii)** $A = ij, B = ij$, **Situation viii)** $A = ij, B = ik$ and **Situation ix)** $A = ij, B = kl$. And it is obvious that under each situation, the equation $\Pr(E|J_i)/\Pr(E|J_9) = 1$ holds.

Table S2: Verification on the $\Pr(E|J_i)/\Pr(E|J_9)$ unification under situation ii)∼iv)

		Situation ii)*		Situation iii)†		Situation iv)‡		Situation v)§	
J_i	Unified formula	Unified	Listed	Unified	Listed	Unified	Listed	Unified	Listed
J_1	$\frac{\mathbb{1}_{ab}\mathbb{1}_{ac}\mathbb{1}_{cd}}{p_a^3}$	0	$\frac{0}{p_i^2p_j^2}$	0	$\frac{0}{2p_i^3p_j}$	0	$\frac{0}{2p_i^2p_jp_k}$	0	$\frac{0}{2p_i^3p_j}$
J_2	$\frac{\mathbb{1}_{ab}\mathbb{1}_{cd}}{p_ap_c}$	$\frac{1}{p_ip_j}$	$\frac{p_ip_j}{p_i^2p_j^2}$	0	$\frac{0}{2p_i^3p_j}$	0	$\frac{0}{2p_i^2p_jp_k}$	0	$\frac{0}{2p_i^3p_j}$
J_3	$\frac{\mathbb{1}_{ab}\left(\mathbb{1}_{ac}+\mathbb{1}_{ad}\right)}{2p_a^2}$	0	$\frac{0}{p_i^2p_j^2}$	$\frac{1}{2p_i^2}$	$\frac{p_ip_j}{2p_i^3p_j}$	0	$\frac{0}{2p_i^2p_jp_k}$	0	$\frac{0}{2p_i^3p_j}$
J_4	$\frac{\mathbb{1}_{ab}}{p_a}$	$\frac{1}{p_i}$	$\frac{p_ip_j^2}{p_i^2p_j^2}$	$\frac{1}{p_i}$	$\frac{2p_i^2p_j}{2p_i^3p_j}$	$\frac{1}{p_i}$	$\frac{2p_ip_jp_k}{2p_i^2p_jp_k}$	0	$\frac{0}{2p_i^3p_j}$
J_5	$\frac{\mathbb{1}_{cd}\left(\mathbb{1}_{ac}+\mathbb{1}_{bc}\right)}{2p_c^2}$	0	$\frac{0}{p_i^2p_j^2}$	0	$\frac{0}{2p_i^3p_j}$	0	$\frac{0}{2p_i^2p_jp_k}$	$\frac{1}{2p_i^2}$	$\frac{p_ip_j}{2p_i^3p_j}$
J_6	$\frac{\mathbb{1}_{cd}}{p_c}$	$\frac{1}{p_j}$	$\frac{p_i^2p_j}{p_i^2p_j^2}$	0	$\frac{0}{2p_i^3p_j}$	0	$\frac{0}{2p_i^2p_jp_k}$	$\frac{1}{p_i}$	$\frac{2p_i^2p_j}{2p_i^3p_j}$
J_7	$\frac{\mathbb{1}_{ac}\mathbb{1}_{bd}+\mathbb{1}_{ad}\mathbb{1}_{bc}}{2p_cp_d}$	0	$\frac{0}{p_i^2p_j^2}$	0	$\frac{0}{2p_i^3p_j}$	0	$\frac{0}{2p_i^2p_jp_k}$	0	$\frac{0}{2p_i^3p_j}$
J_8	$\frac{p_cd_{Ad}+p_dd_{Ac}}{4p_cp_d}$	0	$\frac{0}{p_i^2p_j^2}$	$\frac{1}{2p_i}$	$\frac{p_i^2p_j}{2p_i^3p_j}$	0	$\frac{0}{2p_i^2p_jp_k}$	$\frac{1}{2p_i}$	$\frac{p_i^2p_j}{2p_i^3p_j}$

* $\mathbb{1}_{ab} = \mathbb{1}_{cd} = 1$, $\mathbb{1}_{ac} = \mathbb{1}_{ad} = \mathbb{1}_{bc} = \mathbb{1}_{bd} = 0$, $p_a = p_b = p_i$ and $p_c = p_d = p_j$ under situation ii);
† $\mathbb{1}_{ab} = \mathbb{1}_{ac} = \mathbb{1}_{bc} = 1$, $\mathbb{1}_{cd} = \mathbb{1}_{ad} = \mathbb{1}_{bd} = 0$, $p_a = p_b = p_c = p_i$ and $p_d = p_j$ under situation iii);
‡ $\mathbb{1}_{ab} = 1$, $\mathbb{1}_{cd} = \mathbb{1}_{ac} = \mathbb{1}_{bc} = \mathbb{1}_{ad} = \mathbb{1}_{bd} = 0$, $p_a = p_b = p_i$, $p_c = p_j$ and $p_d = p_k$ under situation iv);
§ $\mathbb{1}_{cd} = \mathbb{1}_{ac} = \mathbb{1}_{ad} = 1$, $\mathbb{1}_{ab} = \mathbb{1}_{bc} = \mathbb{1}_{bd} = 0$, $p_a = p_c = p_d = p_i$ and $p_b = p_j$ under situation v).

Table S3: Verification on the $\Pr(E|J_i)/\Pr(E|J_9)$ unification under situation v)∼vii)

	Situation vi)*		Situation vii)†		Situation viii)‡		Situation ix)§	
J_i	Unified	Listed	Unified	Listed	Unified	Listed	Unified	Listed
J_1	0	$\frac{0}{2p_i^2p_jp_k}$	0	$\frac{0}{4p_i^2p_j^2}$	0	$\frac{0}{4p_i^2p_jp_k}$	0	$\frac{0}{4p_ip_jp_kp_l}$
J_2	0	$\frac{0}{2p_i^2p_jp_k}$	0	$\frac{0}{4p_i^2p_j^2}$	0	$\frac{0}{4p_i^2p_jp_k}$	0	$\frac{0}{4p_ip_jp_kp_l}$
J_3	0	$\frac{0}{2p_i^2p_jp_k}$	0	$\frac{0}{4p_i^2p_j^2}$	0	$\frac{0}{4p_i^2p_jp_k}$	0	$\frac{0}{4p_ip_jp_kp_l}$
J_4	0	$\frac{0}{2p_i^2p_jp_k}$	0	$\frac{0}{4p_i^2p_j^2}$	0	$\frac{0}{4p_i^2p_jp_k}$	0	$\frac{0}{4p_ip_jp_kp_l}$
J_5	0	$\frac{0}{2p_i^2p_jp_k}$	0	$\frac{0}{4p_i^2p_j^2}$	0	$\frac{0}{4p_i^2p_jp_k}$	0	$\frac{0}{4p_ip_jp_kp_l}$
J_6	$\frac{1}{p_i}$	$\frac{2p_ip_jp_k}{2p_i^2p_jp_k}$	0	$\frac{0}{4p_i^2p_j^2}$	0	$\frac{0}{4p_i^2p_jp_k}$	0	$\frac{0}{4p_ip_jp_kp_l}$
J_7	0	$\frac{0}{2p_i^2p_jp_k}$	$\frac{1}{2p_ip_j}$	$\frac{2p_ip_j}{4p_i^2p_j^2}$	0	$\frac{0}{4p_i^2p_jp_k}$	0	$\frac{0}{4p_ip_jp_kp_l}$
J_8	0	$\frac{0}{2p_i^2p_jp_k}$	$\frac{p_i+p_j}{4p_ip_j}$	$\frac{p_ip_j\left(p_i+p_j\right)}{4p_i^2p_j^2}$	$\frac{1}{4p_i}$	$\frac{p_ip_jp_k}{4p_i^2p_jp_k}$	0	$\frac{0}{4p_ip_jp_kp_l}$

* $\mathbb{1}_{cd} = 1$, $\mathbb{1}_{ab} = \mathbb{1}_{ac} = \mathbb{1}_{ad} = \mathbb{1}_{bc} = \mathbb{1}_{bd} = 0$, $p_c = p_d = p_i$, $p_a = p_j$ and $p_b = p_k$ under situation vi);
† $\mathbb{1}_{ac} = \mathbb{1}_{bd} = 1$, $\mathbb{1}_{ab} = \mathbb{1}_{cd} = \mathbb{1}_{ad} = \mathbb{1}_{bc} = 0$, $p_a = p_c = p_i$ and $p_b = p_d = p_j$ under situation vii);
‡ $\mathbb{1}_{ac} = 1$, $\mathbb{1}_{ab} = \mathbb{1}_{cd} = \mathbb{1}_{ad} = \mathbb{1}_{bc} = \mathbb{1}_{bd} = 0$, $p_a = p_c = p_i$, $p_b = p_j$ and $p_d = p_k$ under situation viii);
§ $\mathbb{1}_{ab} = \mathbb{1}_{cd} = \mathbb{1}_{ac} = \mathbb{1}_{ad} = \mathbb{1}_{bc} = \mathbb{1}_{bd} = 0$, $p_a = p_i$, $p_b = p_j$, $p_c = p_k$ and $p_d = p_l$ under situation ix).

4 Instruction of functions of R package *KINSIMU*

4.1 Brief introduction . . . 5
4.2 Install and library the *KINSIMU* package . . . 5
4.3 Functions in the package . . . 5
4.3.1 '*EvaluatePanel*()' . . . 5
4.3.2 '*pairsimu*()' . . . 7
4.3.3 '*pedisimu*()' . . . 9
4.3.4 '*LRparas*()' . . . 10
4.3.5 '*trioPI*()' . . . 12
4.3.6 '*IICAL*()' . . . 13
4.3.7 '*LRhsip*()' . . . 14
4.3.8 '*LRgpgcam*()' . . . 16
4.3.9 '*logLR*()' . . . 18
4.3.10 '*testsimulation*()' . . . 19
4.3.11 '*outputCSV*()' . . . 20
4.4 Data involved . . . 20
4.4.1 *FortytwoSTR* . . . 20
4.4.2 *pediexample* . . . 21

4.1 Brief introduction

The R package *KINSIMU* has been developed using the unified formulae for the assessment and simulation of specific panels in kinship analysis, we hereby present instructions of several functions in the package.

4.2 Install and library the *KINSIMU* package

The authors have uploaded the package to CRAN, making it able to be install directly with the following code:

```
install .package("KINSIMU")
```

Otherwise, the repository file "KINSIMU.0.1.1.tar.gz" is provided as File S2 of this study, and the package can be installed using the subsequent code after downloading it:

```
utils ::: menuInstallLocal ()
```

After installing the package, it can be loaded with the following code:

```
library ("KINSIMU")
```

4.3 Functions in the package

In this section, we will introduce the functions contained in the *KINSIMU* package. Each function introduction will consist of four parts: (i) input arguments; (ii) output values; (iii) detailed description of the function's process, presented as algorithms. Code and methods for key steps will be labeled with †, ‡, # and § in the algorithms, followed by detailed explanations. (iv) Examples of the function.

There would be several common abbreviations in this section:
nl: number of loci in the panel, used in '*EvaluatePanel*()', '*testsimulation*()' and '*logLR*()' functions;
ss: sample size to be simulated in '*pairsimu*()','*pedisimu*()' and '*testsimulation*()' functions;
$\boldsymbol{n_a}$: number of alleles on a locus;
np: number of individual to be simulated in '*pedisimu*()' function;
stop in algorithms: Terminate the function without executing any subsequent code.

4.3.1 '*EvaluatePanel*()'

The function would transfer the input allele frequency data (in a .csv file or a data frame) into form usable for other functions and then calculate several population parameters based on the frequency data for each marker.

Input arguments 4 input arguments are needed for the function, one of which is optional.

- **type**: The type of input data, there are 2 acceptable type of input for the argument:
  - '*csv*': a .csv file containing the allele frequency data in the panel. In such a file, cell 'A1' should be 'allele' and the other cells in the first row should contain the names of each marker. The names of all detected allele types in the panel should be listed in the first column and the corresponding frequency data on each marker should be contained in the cells according to marker names and allele names. If the input argument '**raremode**' is set as '*ISFG*' or '*1/2N*' and the input argument '**Nind**' is set as '*lastrow*', the sample size in the population survey should be listed in the last row per marker;
  - '*df*': a data frame with the similar structure with the aforementioned .csv file, except that the word 'allele' and marker names should be the column names;
- **strpath**: The pathway of the .csv file or the name of the data frame, according to the set of argument **type**.
- **raremode**: The mode of calculation method of rare allele frequency at each locus. There are 3 acceptable type of input for the argument
  - '*ISFG*': The default input, rare allele frequency data is calculated based on the population survey results, i.e., $p_{rare} = (n_a + 1)\big/(2\mathcal{N} + 1)$ if n_a and $\mathcal{N}$ denote the number of allele types detected and the sample size, respectively;
  - '*MAF*': The minimum allele frequency is taken as the rare allele frequency;
  - '*1/2N*': The minimum allele frequency a population survey with a sample size $\mathcal{N}$ can provide is taken as the rare allele frequency;
  - <*numeric*>: A number set as the rare allele frequency, the function would report error if the number > 1;
- **Nind**: (Optional) Mode of sample size data presentation, only need when **raremode** is set as '*ISFG*' or '1/2N'. There are 2 acceptable types of input for the argument:
  - '*lastrow*': The sample size data is given in the last row of the .csv file;
  - <*integer*>: An integer number can be input and taken as the sample size on all markers.
- **Th**: The threshold for the difference in allele frequency sum at a locus with 1, to detect data error from rounding error when the frequency sum does not equal 1. Loci exceeding this threshold will be excluded from the calculation.

Output values A list of 4 vectors would be output:

- **afmatrix**: A list of *nl* data frames that hold information of the allele frequencyfor each marker. Each data frame is structured with a single column and a row count of n_a. The row names of the data frames list the names of the alleles.
- **rare**: A data frame containing rare allele frequencies on each marker;
- **indicators**: A data frame containing forensic parameters for each marker, which would be detailed introduced;
- **panelpara**: A data frame containing population parameters for the whole panel, with the form of $\log_{10}$(1-paramter) to avoid displaying the parameters as 1 because they are too close to 1.

Detailed description Upon performing an analysis of a specified .csv file or data frame, this function extracts allele frequency data at different loci and evaluate the population parameters for the specific kit using the provided data. The complete procedure is detailed in Algorithm 1.

Algorithm 1: EvaluatePanel()

```
Input basic data (allelefreq) from a .csv file or a data frame pointed by argument 'strpath', according to 'type' set;
Conduct formal checks on basic data, stop if any error exists;
if Nind='lastrow' then
    Take the last row of allelefreq data frame as n_of_indi data frame;
    Take the rest rows as allelefreq data frame;
else if Nind=<integer> then
    Repeat the Nind value as n_of_indi data frame;
end
Delete loci for which the difference between the sum of allele frequencies and 1 exceeds Th;
Calculate population parameters for each locus and generate the indicators data frame;          /* † */
Calculate combined population parameters for the whole panel and generate the panelpara data fram;
Extract/calculate rare frequency and generate the rare data frame according to raremode;          /* ‡ */
Divide the allelefreq data frame into nl single data frames and delete rows with 0 values for each;
Integrate the nl data frames into a list named afmatrix;
Integrate the four output vectors into a list Result and output the list.
```

† The **indicators** data frame contains 12 population parameters computed for each locus:

Table S4: Parameters presented in **indicator** data frame

No.	Name	Note	Equation
1	n_a	The number of allele types on a marker	–
2	H_u	The unadjusted heterozygosity	$H_u = 1 - S_2{}^*$
3	H_a	The adjusted heterozygosity	$H_a = H_u \times \frac{n_a}{n_a - 1}$
4	MAF	The minimum allele frequency	–
5	PM	The probability of randomly match	$PM = 2S_2^2 - S_4$
6	DP	The discrimination power	$DP = 1 - PM$
7	PED	Probability of exclusion (PE) in duo paternity cases	$PED = 1 - 4S_2 + 4S_3 + 2S_2^2$
8	PET	PE in trio paternity cases	$PET = 1 - 2S_2 + S_3 + 2S_4 - 3S_5 - 2S_2^2 + 3S_2S_3$
9	PEDD	PE in double doubt parentage cases	$PEDD = 1 + 4S_4 - 4S_5 - 3S_6 - 8S_2^2 + 2S_3^2 + 8S_2S_3$
10	RGE	Mean Power of Random Grandparents Excluded**	$RGE = 1 - 4S_2 + 6S_3 - 17S_5 + 28S_6 - 15S_7 - 4S_2^2 + 18S_2S_3 - 16S_2S_4 + 5S_2S_5 - 12S_3^2 + 10S_3S_4$
11	RGENM	RGE without the assistant with the mother	$RGENM = 1 - 8S_2 + 16S_3 - 26S_4 + 30S_5 - 15S_6 + 12S_2^2 - 24S_2S_3 + 8S_2S_4 + 6S_3^2$
12	PIC	polymorphism information content	$PIC = 1 - S_2 - S_2^2 + S_4$

* S_x represents the sum of all frequencies on a locus to the power of x, i.e., $S_x = \sum_i p_i^x$;

** RGE is calculated for grand-parentage identification involving four participants: a child, the child's biological mother and a couple of individuals who are alleged as the child's grandparents, i.e., the parents of the child's father.

‡ The data frame **panelpara** includes 6 types of total (T) or cumulative (C) population parameters are calculated for the whole panel: TDP, CPED, CPET, CPEDD, CRGE, CRGENM. The calculation method of these parameters are similar, if set specific parameter on the ith marker as $\mathcal{P}_i$, the total parameter $\mathcal{TP}$, then

$$\mathcal{TP} = 1 - \prod_i \mathcal{P}_i \tag{S5}$$

Examples Two examples are given based on a .csv file generated by '*outputCSV*()' function. The two examples would result in a same output, which is included in the package as data "*FortytwoSTR*".

```
#A .csv  file  can be output with  FortytwoSTR data
path<-tempdir()
outputCSV(FortytwoSTR,file.path(path,'data.csv'))
#Example 1, 'df'  type,  by read the csv  file   into  a data frame
 allele _data <- read.csv( file  =  file .path(path,'data.csv'),  header  = TRUE)
STR42<- EvaluatePanel(type = 'df', strpath  =  allele _data,raremode = "ISFG",Nind = "lastrow")
#Example 2, 'csv' type,  the  same evaluation can be done by directly input  the  csv  file
STR42_2 <- EvaluatePanel(type =  'csv', strpath   =  file .path(path,'data.csv'),
raremode = "ISFG",Nind = "lastrow")
#The data "FortytwoSTR" is generated with these codes.
```

4.3.2 '*pairsimu*()'

The function would generate genotype combinations of multiple individual pairs with specific relationships on an autosomal marker, ignoring mutaion.

Input arguments 4 input arguments are needed for the function:

- **af**: A data frame of 1 column containing the allele frequency data on the marker with the allele names being the row names and 'Freq' as column name, which can be generated with function ''*EvaluatePanel*()'' (e.g., 'af=FortytwoSTR$afmatrix[[1]]') or input with function '*data.frame*(Freq=c(· · ·))' directly;
- **ss**: The sample size of the simulation;
- **delta**: The distribution of IBD or Jacquard coefficient of the target relationship, which should be input as a single row of data with function '*c*()', the coefficients should be input in order of '$\kappa_0 \to \kappa_2$' or '$\Delta_1 \to \Delta_9$';
- **allelename**: The output format setting, There are 2 acceptable types of input for the argument:
  - *FALSE*: The default set, to output the row number of the alleles in the **af** matrix;
  - *TRUE*: To output allele names, i.e., the row names in the **af** matrix.

Output value A data frame of 4 columns and ss rows would be output, which denote the genotype combinations of each pair simulated, the first 2 columns denote individual A and the other 2 individual B.

Detailed description A directly generating strategy is applied in this function:

Algorithm 2: pairsimu()

```
Construct the result data frame in the aforementioned form;
if The length of delta is 3 (non-inbred cases) then
    Randomly generate the genotypes (in form of allele position) of ss individual As;      /* † */
    Generate ss random numbers 1~4 according to delta set;                                 /* ‡ */
    Generate genotypes of ss indivdiual Bs;
else if The length of delta is 9 (inbred cases) then
    Randomly generate the first alleles (in form of allele position) of ss individual As;  /* † */
    Generate ss random numbers 1~9 according to delta set;                                 /* # */
    Generate the second alleles of ss individual As and genotypes of ss indivdiual Bs;
else
    Report the error of delta setting and stop;
end
if allelename='TRUE' then
    Translate the allele positions into allele names;                                      /* § */
end
Output the result data frame.
```

† These alleles can be randomly generated with '*sample*()' function, take the 1st allele of individual A as example:

```
pop<-1:nrow(af)
 results $A1<-sample(x=pop,size=ss,replace=TRUE,prob=af$Freq)
```

‡ For outbred relationships with $\kappa = \{\kappa_0, \kappa_1, \kappa_2\}$, there are 4 types of possible IBD genotype of individual B, (i) $x_I y_I$, (ii) $b_I x_I$, (iii) $a_I x_I$, and (iv) $a_I b_I$, the corresponding probabilities of which are κ_0, $\kappa_1/2$, $\kappa_1/2$ or κ_2, respectively. Among these IBD genotypes, $b_I x_I$ can be written as $x_I b_I$, which would not affect any of the following calculations. Thus, the 1st allele of individual B would be IBD to the 1st of individual A if situation (iii) or (iv) happens, while his/her 2nd allele would be IBD to the 2nd of individual A if situation (ii) or (iv) happens. In other words, the genotypes of individual Bs can be generated according to '**delta**' value with the following code:

```
# Randomly generate individual A's alleles
 results $A1<-sample(x=pop,size=ss,replace=TRUE,prob=af$Freq)
 results $A2<-sample(x=pop,size=ss,replace=TRUE,prob=af$Freq)
# Generate random numbers (RN), with value of 1:4 according to delta distribution
# Each number stands for a situation of  individual  B's  IBD genotype
RN<-sample(x=1:4,size=ss,replace=TRUE,prob=c(delta[1],delta[2]/2, delta [2]/2, delta [3]) )
# T1: numbers lager than 2 in RNs, i.e.,  situation  ( iii )  or  (iv)  happens
T1<-as.double(RN>2)
# T2: even numbers in RNs, i.e.,  situation  ( ii )  or  (iv)  happens
T2<-as.double(RN%%2==0)
# Transfer  situations  into  individual  B's  alleles
 results $B1<-sample(x=pop,size=ss,replace=TRUE,prob=af$Freq)*(1-T1)+results$A1*T1
 results $B2<-sample(x=pop,size=ss,replace=TRUE,prob=af$Freq)*(1-T2)+results$A2*T2
```

For inbred relationships, the two alleles of individual A may be IBD to each other, i.e., only the first alleles of each individual A can be generated randomly based on the allele frequency data and other 3 alleles should be generated according to the Δ distribution, which is more complex compared to outbred cases. The corresponding code can be written as follows:

```
 results $A1<-sample(x=pop,size=ss,replace=TRUE,prob=af$Freq)
RN<-sample(x=1:11,size=ss,replace=TRUE,prob=c(delta[1],delta [2], delta [3], delta [4], delta [5]/2, delta [5]/2, delta [6], delta [7], delta [8]/2, delta [8]/2, delta [9]) )
T0<-as.double(RN>4)
T1A<-as.double(RN%in%c(1,3,5,8,9))
T1B<-as.double(RN==6)
T2A<-as.double(RN%in%c(1,8,10))
T2B<-as.double(RN%in%c(2,5,6,7))
 results $A2<-sample(x=pop,size=ss,replace=TRUE,prob=af$Freq)*T0+results$A1*(1-T0)
 results $B1<-sample(x=pop,size=ss,replace=TRUE,prob=af$Freq)*(1-T1A-T1B)+results$A1*T1A+results$A2*T1B
 results $B2<-sample(x=pop,size=ss,replace=TRUE,prob=af$Freq)*(1-T2A-T2B)+results$A2*T2A+results$B1*T2B
```

§ If argument **allelename** is set as TRUE, the allele position can be translated to allele names with the following codes:

```
an<-as.data.frame(as.numeric(row.names(af)))
 results $A1<-an[results$A1,]
 results $A2<-an[results$A2,]
 results $B1<-an[results$B1,]
 results $B2<-an[results$B2,]
```

Example Three examples are given, simulating 3 types of individual pairs based on the 1st STR in the 42 ones in '*FortytwoSTR*' data set, setting ss=10,000:

```
# Extract  allele  frequency on the locus
af = FortytwoSTR$afmatrix[[1]]
# simulating  10,000 unrelated pairs
```

```
a<-pairsimu(af = af, ss = 10000, delta = c (1,0,0) , allelename = FALSE)
# simulating 10,000 parent-child pairs
b<-pairsimu(af = af, ss = 10000, delta = c (0,1,0) , allelename = FALSE)
# simulating 10,000 full-sibling pairs
c<-pairsimu(af = af, ss = 10000, delta = c (0.25,0.5,0.25) , allelename = FALSE)
```

4.3.3 ‘*pedisimu*()’

The function would genotype combinations of multiple pedigrees with specific relationships on an autosomal marker.

Input arguments 7 input arguments are needed for the function, 2 of which are optional.

- **af**: A data frame of similar to **af** in ‘*pairsimu*()’ function;
- **ss**: The sample size of the simulation;
- **pedi**: a data frame containing the pedigree structure information, with 3 columns (“Person”, “Father” and “Mother”) and *np* rows;
- **random_name**: The name of random individual, with a default of "RI";
- **muf**: (Optional) father-child mutation rate, with default set of 0, only need if **allelename** set as *TRUE*;
- **mum**: (Optional) mother-child mutation rate, with default set of 0, only need if **allelename** set as *TRUE*;
- **allelename**: Similar to **allelename** in ‘*pairsimu*()’ function.

Output value A data frame of ss rows and 2×*np* columns. Each pair of columns contains alleles of an individual, with the individuals sorted in the same order as in the **pedi** data frame.

Detailed description Genotype data is generated sequentially according to Algorithm 3:

Algorithm 3: pedisimu()

```
Conduct formal checks on the data frame pedi, and stop if any error exists;
Extract np as the row number of pedi data frame;
Construct result data frame with ss rows and 2×np columns;
for i in 1:np do
    if There is no individual in “Person” column named identical as the father of ith individual then
        Randomly generate the ith individual’s paternal allele according to allelename set;   /* † */
    else
        Set f=the row number of the ith individual’s father in “Person” column;
        if f≥i then
            Report error “Father of the ith individual should be defined before him/her” and stop;
        end
        Generate the ith individual’s paternal allele from the fth individual’s genotype;   /* ‡ */
        if muf>0 then
            Randomly change the alleles according to muf set;   /* # */
        end
    end
    Generate the maternal allele of the ith individual with similar process considering mum setting;
end
```

† Alleles without parent setting can be generated with ‘*sample*()’ function, according to the **allelename** set. Take the paternal allele of the ith individual as example:

```
if (isTRUE(allelename)) { results [,2*i-1]<-sample(x=as.numeric(row.names(af)),size=ss,replace=TRUE,prob=af$Freq)
} else {pop<-1:nrow(af)
  results [,2*i-1]<-sample(x=pop,size=ss,replace=TRUE,prob=af$Freq)
}
```

‡ If father or mother of the ith individual is set, the corresponding allele can be generated according to the genetic law, i.e., the two alleles of the father or mother have equivalent probabilities to be inherited. Take the paternal inheritance of the offspring as example:

```
RN<-sample(x=c(0,1),size=ss,replace=TRUE,prob=c(0.5,0.5))
 results [,2*i-1]<-results [,2*f-1]*RN+results[,2*f]*(1-RN)
```

To perform mutation, the original alleles of the offspring would be added by a random number within set {-1,0,1} according to the mutation rate:

```
if (muf>0) { results [,2*i-1]= results [,2*i-1]+sample(x=c(-1,0,1), size=ss, replace=TRUE,prob=c(muf/2,1-muf,muf/2))}
```

Example An example is given to simulate 10,000 first cousin pedigree (as listed in data '**pediexample**' included in the package), based on the first locus in data '*FortytwoSTR*':

```
pedi<-pediexample
af<-FortytwoSTR$afmatrix[[1]]
pedisimu(af=af, ss=10000,pedi=pedi)
```

4.3.4 '*LRparas*()'

The function would count or calculate parameters used in the calculation of different pairwise LR.

Input arguments 7 input arguments are needed for the function

- **AB**: A data frame of 4 columns containing the genetic information of the participants, each row denote a single pair, and the 4 columns containing alleles *a*, *b*, *c* and *d*, respectively. The data frame can be generated with functions ''*pairsimu*()'' or ''*pedisimu*()'', or can be directly input with function '*data.frame*()';
- **af**: Allele frequency data, There are 2 acceptable types of input for the argument:
  - *NULL*: The default set;
  - <*data frame*>: input a data frame similar to **af** in '*pairsimu*()' function;
- **rare**: Rare frequency data, there are 2 acceptable types of input for the argument:
  - *NULL*: The default set;
  - <*numeric*>: A number of rare frequency;
- **stepwisePI**: Whether stepwise mutation model should be considered when calculating paternity index, there are 2 acceptable types of input for the argument:
  - *FALSE*: The default set, take mutation rate as PI if no allele is shared between the two individuals;
  - *TRUE*: Calculate PI considering stepwise mutation model in paternity tests;
- **bred**: Whether $J_1 \sim J_6$ should be calculated, there are 2 acceptable types of input for the argument:
  - *FALSE*: The default set, output 2 ratios: (i) $\Pr(E|J_8)/\Pr(E|J_9)$, i.e., paternity index which is labeled as '*PInomu*' in the output data frame; (ii) $\Pr(E|J_7)/\Pr(E|J_9)$, i.e., likelihood ratio in personal identification which is labeled as '*LRid*' in the output data frame;
  - *TRUE*: If so, the other 6 ratios in Eq. (15) would be output, labeled as '*FD1*' $\rightarrow$ '*FD6*', i.e., factors of $\Delta_1 \rightarrow \Delta_6$;
- **mu**: The mutation rate if paternity index is calculated, with a default of 0.002;
- **allelename**: The data type of **AB** data frame, there are 2 acceptable types of input for the argument:
  - *FALSE* To treat the values in **AB** data frame as row numbers in **af** data frame;
  - *TRUE* To treat the values in **AB** data frame as allele names (row names) in **af** data frame.

Output value A data frame of **ss** rows and multiple columns, containing the combined identity by state (CIBS) score, the 8 or 2 ratios needed in Eq. (15) or Eq. (16) in the main text, respectively, as well as PI considering mutation.

Detailed description The function is performed as Algorithm 4. It can be seen that all these parameters are calculated simultaneously, as a result, LR of the ss pairs can be calculated simultaneously on a single marker.

Algorithm 4: LRparas()

Conduct formal checks on the data frame **AB**, and **stop** if any error exists;
Calculate $\mathbb{1}_{ac}$, $\mathbb{1}_{ad}$, $\mathbb{1}_{bc}$ and $\mathbb{1}_{bd}$ based on **AB** data frame; /* † */
Calculate IBS score and set as the first column of the output result data frame; /* ‡ */
if *(**af**='NULL') OR (**allelename**='TRUE' AND **rare**=1NULL')* **then**
  if ***stepwisePI*** *or* ***bred*** *is set as TRUE* **then**
    Report error "Please input the frequency data" and **stop**;
  end
  Output the result data frame; /* No frequency data for LR calculation */
else
  Extract p_c and p_d; /* # */
  Calculate LRid and PInomu (PI not considering mutation) as the 2nd and 3rd columns of the result data frame, according to Eq. (16) in main text;
  if *All values in PInomu column >0* **then**
    Set PImu=PInomu as the 4th column of the result data frame;
  else if ***stepwisePI*** *is set as TRUE* /* § */ **then**
    Calculate the 4 absolute differences between the two indivdiual's alleles ($a \to c$, $a \to d$, $b \to c$, and $b \to d$), transfer non-integer-step mutations into numbers larger than 10000;
    Choose the minimum in the 4 differences as the minimum mutation step (s);
    Calculate d_{Ac} and d_{Ad} as the dosage of alleles that can mutate into c and d with s steps;
    if s=0 **then**
      PImu=PInomu;
    else if s>10000 **then**
      PImu=μ, which is defined by input argument **mu**;
    else
      $PImu = \mu \times 10^{1-s} \times \left(\frac{d_{Ac}}{8p_c} + \frac{d_{Ad}}{8p_d}\right)$;
    end
  else
    Calculate PImu, by taking **mu** as PI when no allele is shared between the two individuals;
  end
  if ***bred*** *is set as TRUE* **then**
    Extract p_a, calculate $\mathbb{1}_{ab}$ and $\mathbb{1}_{cd}$;
    Calculate the other 6 ratios in Eq. (15) as the 5th∼10th columns of the result data frame;
  end
  Output the result data frame
end

† The $\mathbb{1}$ parameters can be calculated using '*as.duoble*()' function, take $\mathbb{1}_{ac}$ as example:

```
ac<-as.double(AB[,1]==AB[,3])
```

‡ The IBS score is a parameter evaluating the similarity of two individual's genotypes. It equals to 2 if the genotypes are identical (i.e., $\mathbb{1}_{ac}\mathbb{1}_{bd} + \mathbb{1}_{bc}\mathbb{1}_{ad} > 0$), to 0 if there is no allele sharing between them (i.e., $\mathbb{1}_{ac} + \mathbb{1}_{bd} + \mathbb{1}_{bc} + \mathbb{1}_{ad} = 0$), and to 1 otherwise. Thus, the score can be calculated with following codes:

```
ibs=as.double(ac+ad+bc+bd>0)+as.double(ac*bd+ad*bc>0)
```

The frequency value can be extracted from **af** data frame according to **allelename** setting, then, frequency of rare alleles should be replaced by **rare** value. Take p_c as example:

```
if (isTRUE(allelename)) {pc<-af[as.character(AB[,3]),]} else {pc<-af[AB[,3],]}
pc[is.na(pc)]<-rare
```

§ When using STR markers in parentage testing, the relatively high mutation rate of these markers leads to a greater likelihood that a true parent-child pair may not share any allele. In such cases, it is important to calculate the PI considering mutation in order to prevent the erroneous exclusion of parentage. Replication slippage is the primary cause of mutation for STR markers, and a model known as the "stepwise mutation model" has been developed, considering that the probability of mutating s+1 steps as being one-tenth of the probability of mutating s steps. Thus, the probability of allele x mutated to allele x+s equal to $0.5 \times \mu \times \left(\frac{1}{10}\right)^{s-1}$, where the first "0.5" denote the probability of mutating longer or shorter. And the calculation of PI considering mutation can be performed with the following codes:

```
# Calculate the absolute difference between the two individual's alleles, take abs(a-c) as example
if (is.TRUE(allelename)) {
  acm=abs(AB[,1]-AB[,3])+abs(AB[,1]-AB[,3])%%1*100000
} else {
  an<-as.data.frame(as.numeric(row.names(af)))
```

```
  acm=abs(an[AB[,1],]-an[AB[,3],])+abs(an[AB[,1],]-an[AB [,3],]) %%1*100000
}
# Choose the minimum mutation steps
steps<-pmin(acm,adm,bcm,bdm)
# dc and dd calculation
dc<-as.double(acm==steps)+as.double(bcm==steps)
dd<-as.double(adm==steps)+as.double(bdm==steps)
#PImu calculation
PImu<-as.double(steps==0)*para$PInomu+ # ignore mutation if step=0, i.e., there is at least one sharing allele between the two indiviudals
  as.double(steps>10000)*mu+ # take mutation rate as PI if there is no integer step of mutation
  as.double(steps>0 & steps<10000)*mu*10^(1-steps)*(dc/pc+dd/pd)/8 # step wise model of mutation
```

Example An example is given, simulating and calculating parameters for 10,000 parent-child pairs

```
af = FortytwoSTR$afmatrix[[1]]
AB = pairsimu(af = af, ss = 10000, delta = c(0,1,0) , allelename = FALSE)
LRelements<-LRparas(AB=AB, af=af, rare=FortytwoSTR$rare[1],allelename=FALSE,stepwisePI=TRUE,bred=TRUE)
```

The following 4 functions are utilized to compute more intricate LR for specific identification across multiple cases on a single marker:

4.3.5 ‘*trioPI*()’

The function would Calculate LR in standard trio cases, where 3 participants being available, a child (C), one of his/her confirmed parent (TP), as well as an individual who is unrelated to TP and alleged to be specific relative of the child (AR). Usually, AR is the alleged father of C, as in standard trio paternity testing. Null hypothesis, i.e., that the alleged participant is unrelated to the child, is taken as Hd. Inbreeding factors are not considered.

Input arguments 9 input arguments are needed for the function

- **AR**: A data frame of 2 columns containing the genetic information of ARs;
- **C**: A data frame of 2 columns containing the genetic information of Cs;
- **TP**: A data frame of 2 columns containing the genetic information of ARs;
- **af**: A data frame similar to **af** in ‘*pairsimu*()’ function;
- **rare**: The frequency of rare alleles, which is similar to **rare** in ‘*LRparas*()’ function;
- **allelename**: The data type of **AR**, **C** and **TP** data frames, similar to **allelename** in ‘*LRparas*()’ function;
- **muAtoC**: Mutation rate from AR to C, if AR is alleged to be a parent of C. With a default set of 0.002, please note that mistakes would be introduced if the mutation rate is larger than 0.2;
- **muTtoC**: Mutation rate from TP to C, with a default of 0.002/3.5, please note that mistakes would be introduced if the mutation rate is larger than 0.2;
- **kappa1**: The κ_1 between AR and C under Hp, with a default set of 1, i.e., trio paternity testing.

Output value A data frame of **ss** rows and 1 columns would be output, containing the $\log_{10}$LR of each case.

Detailed description The function is performed as Algorithm 5.

Algorithm 5: trioPI()

Conduct formal checks on the 3 genotype data frames, and **stop** if any error exists;
Extract p_c and p_d with similar method to code in “#” of ‘*LRparas*()’ function;
Calculate the four $\boldsymbol{d}$ parameters in Eq. (25) of the main text, each as the summation of two $\mathbb{1}$ parameters calculated similar to code in “†” of ‘*LRparas*()’ function;
if ***kappa1**<1* **then**
  if $d_{TPc} + d_{TPd} > 0$ *for all cases* **then**
    Calculate results directly according to Eq. (25) in the main text:
    $LR = \frac{\kappa_1 d_{TPc} d_{ARd} + \kappa_1 d_{TPd} d_{ARc}}{2 d_{TPc} p_d + 2 d_{TPd} p_c} + (1 - \kappa_1)$
  else
    Calculate results considering mutation from TP to C;
  end
else
  if $d_{TPc} d_{ARd} + d_{TPd} d_{ARc} > 0$ *for all cases* **then**
    Calculate results directly according to Eq. (27) in the main text:
    $PI_{trio} = \frac{d_{Mc} d_{AFd} + d_{Md} d_{AFc}}{2 d_{Mc} p_d + 2 d_{Md} p_c}$ /* kappa=1, i.e., trio paternity testing */
  else
    Calculate results condidering mutations from both TP and AR to C.
  end
end

Example Examples of two type of identifications are given: trio paternity test and avuncular test with the assistance of the child's mother:

```
# Three types of pedigrees are simulated: pedi1: father−mother−child; pedi2: random male−mother−child; and pedi3: uncle−mother−child
pedi1 <− data.frame(Person=c("F","M","C"),Father=c("RI","RI","F"),Mother=c("RI","RI","M"))
pedi2 <− data.frame(Person=c("F","M","C"),Father=c("RI","RI","RI"),Mother=c("RI","RI","M"))
pedi3 <− data.frame(Person=c("GF","GM","AR","F","M","C"),
Father=c("RI","RI","GF","GF","RI","F"),
Mother=c("RI","RI","GM","GM","RI","M"))
# Two types of LRs are calculated: PI_1 and PI_2: Paternity index for pedi1 and pedi 2; AI_1 and AI_2: Avuncular index in trio cases for pedi2 and pedi3.

PI_1=PI_2=AI_1=AI_2=data.frame(Log10CLR=rep(0,10000))
# Simulation are carried out based on the frequency data of the 42 STRs in FortytwoSTR dataset setting sample size as 10,000
for (i in 1:42) {
    Genotype1<−pedisimu(af = FortytwoSTR$afmatrix[[i]], ss = 10000,pedi = pedi1)
    PI_1<−PI_1+trioPI(AR=Genotype1[,1:2],TP=Genotype1[,3:4],
                    C=Genotype1[,5:6],af=FortytwoSTR$afmatrix[[i]],
                    rare=FortytwoSTR$rare[i])
    Genotype2<−pedisimu(af = FortytwoSTR$afmatrix[[i]], ss = 10000,pedi = pedi2)
    PI_2<−PI_2+trioPI(AR=Genotype2[,1:2],TP=Genotype2[,3:4],
                    C=Genotype2[,5:6],af=FortytwoSTR$afmatrix[[i]],
                    rare=FortytwoSTR$rare[i])
    AI_2<−AI_2+trioPI(AR=Genotype2[,1:2],TP=Genotype2[,3:4],
                    C=Genotype2[,5:6],af=FortytwoSTR$afmatrix[[i]],
                    rare=FortytwoSTR$rare[i],kappa1=0.5)
    Genotype3<−pedisimu(af = FortytwoSTR$afmatrix[[i]], ss = 10000,pedi = pedi3)
    AI_1<−AI_1+trioPI(AR=Genotype3[,5:6],TP=Genotype3[,9:10],
                    C=Genotype3[,11:12],af=FortytwoSTR$afmatrix[[i]],
                    rare=FortytwoSTR$rare[i],kappa1=0.5)
  }
#histogram of the final results
xmin1<−floor(min(min(PI_1$Log10CLR),min(PI_2$Log10CLR)))
xmax1<−ceiling(max(max(PI_1$Log10CLR),max(PI_2$Log10CLR)))
xmin2<−floor(min(min(AI_1$Log10CLR),min(AI_2$Log10CLR)))
xmax2<−ceiling(max(max(AI_1$Log10CLR),max(AI_2$Log10CLR)))
par(mfrow = c(2, 2))
hist(PI_1$Log10CLR,xlab = expression(log[10]~CPI),main = "True parentage cases",
     xlim = c(xmin1,xmax1), col = "blue")
hist(AI_1$Log10CLR,xlab = expression(log[10]~CAI),main = "True avuncular cases",
     xlim = c(xmin2,xmax2), col = "blue")
hist(PI_2$Log10CLR,xlab = expression(log[10]~CPI),main = "False pedigree in parentage cases",
     xlim = c(xmin1,xmax1), col = "red")
hist(AI_2$Log10CLR,xlab = expression(log[10]~CAI),main = "False pedigree in avuncular cases",
     xlim = c(xmin2,xmax2), col = "red")
```

4.3.6 '*IICAL*()'

The function would calculate incest index (II_{φ}), i.e., the ratio for a parent-child pair between the probability that the child's other parent is a relative of the present parent to the probability that the child's parents are unrelated.

Input arguments 6 input arguments are needed for the function

- **Parent**: A data frame of 2 columns containing the genetic information of the present parent;
- **Child**: A data frame of 2 columns containing the genetic information of Cs;
- **af**: A data frame similar to **af** in '*pairsimu*()' function;
- **rare**: The frequency of rare alleles, which is similar to **rare** in '*LRparas*()' function;
- **allelename**: The data type of **Parent** and **Child** data frames, similar to **allelename** in '*LRparas*()' function;
- **phi**: The kinship coefficient φ between the two parents under Hp, with a default set of 0.25, i.e., assuming that they are parent-child pair or full-siblings.

Output value A data frame of **ss** rows and 2 columns would be output, containing two parameters used in such cases: (i) Ngs: the state of genotype similarity (gs) between the parent-child pair, i.e., whether both of the child's two alleles can be inherited from the present parent; (ii) the $\log_{10} II_{\varphi}$ of each case.

Detailed description The function is performed as Algorithm 6.

Algorithm 6: IICAL()

1. Conduct formal checks on the 2 genotype data frames, and **stop** if any error exists;
2. Extract or calculate the two ***d*** parameters and two ***p*** parameters in Eq. (20) of the main text with codes similar to corresponding ones in '*LRparas*()' function: $II_{\varphi} = \frac{2\varphi d_{Ac} d_{Ad}}{d_{Ac} p_d + d_{Ad} p_c} + (1 - 2\varphi)$;
3. Calculate Ngs with code "Ngs<-1-as.double(dc*dd==0)";
4. Calculate II_{φ} with code "IIphi<-log10(2*phi*dc*dd/(dc*pd+dd*pc)+1-2*phi)";
5. Translate the error values (for with the parent cannot provide any of the child's alleles) into 1-2φ.

Note that if there is no allele sharing between the two participants, II_{φ} cannot be calculated directly according to Eq. (20) in the main text due to the 0 value of the denominator. In this case, $(1-2\varphi)$ would be the output.

Example Two examples are given, simulating and calculating $CII_{0.25}$ based on the 42 STRs in *FortytwoSTR* dataset for 10,000 mother-child pairs when Hp and Hd in the formula is true, respectively.

```
# Construct the pedi data.frame for incest cases
pedi<-data.frame(Person=c("F","M","C"),Father=c("RI","F","F"),Mother=c("RI","RI","M"))
II_1=II_2=data.frame(Ngs=rep(0,10000),IIphi=rep(0,10000))
for (i in 1:42) {
  # Simulate 10,000 mother-child pairs with father-daughter incest with pedisimu() function
  Genotype1<-pedisimu(af = FortytwoSTR$afmatrix[[i]], ss = 10000,pedi = pedi)
  II_1<-II_1+IICAL(Parent = Genotype1[,3:4],Child = Genotype1[,5:6], af=FortytwoSTR$afmatrix[[i]],
  rare=FortytwoSTR$rare[i][1,1], phi=0.25)
  #Simulate 10,000 non-inbred mother-child pairs with pairsimu() function
  Genotype2<-pairsimu(af = FortytwoSTR$afmatrix[[i]], ss = 10000,delta = c(0,1,0) ,allelename = FALSE)
  II_2<-II_2+IICAL(Parent = Genotype2[,1:2],Child = Genotype2[,3:4], af=FortytwoSTR$afmatrix[[i]],
  rare=FortytwoSTR$rare[i][1,1], phi=0.25)
}
# histograms of CII distributions in the two groups
xmin<-floor(min(min(II_1$IIphi),min(II_2$IIphi)))
xmax<-ceiling(max(max(II_1$IIphi),max(II_2$IIphi)))
par(mfrow = c(1, 2))
hist(II_2$IIphi,xlab = expression(log[10]~CII),main = "Non-inbreed cases",
xlim = c(xmin,xmax), col = "red")
hist(II_1$IIphi,xlab = expression(log[10]~CII),main = "Inbreed cases",
xlim = c(xmin,xmax), col = "blue")
```

4.3.7 '*LRhsip*()'

The function would calculate LR for cases in which a pair of siblings (labeled as A and B, with genotype *ab* and *cd*, respectively) and one of their identical parent participated (labeled as P with genotype *ef*). H_p and H_d are set as "the other parents of the two siblings are specific related" and "the other parents of them are unrelated", respectively. Inbreeding factors are not taken into consideration.

Derivation of LR in the identification Regard sibling B as individual B, and the other two individuals as R in Eq. (21) of the main text. Similar to "standard" non-inbred trio cases in section 2.4.2 of the main text, the relationship between P and A would remain unchanged in both hypotheses if not considering inbreeding factors, thus, similarly

$$LR = \frac{\sum_j \left[\Pr\left(B \equiv \mathcal{G}_j \middle| A, P, H_p\right) \times \Pr\left(\mathcal{G}_j \equiv cd\right)\right]}{\sum_k \left[\Pr\left(B \equiv \mathcal{G}_k \middle| A, P, H_d\right) \times \Pr\left(\mathcal{G}_k \equiv cd\right)\right]} \tag{S6}$$

Under H_d, the non-participated parent of sibling B is unrelated to both P and sibling A, the allele he/she passed to sibling B must be x_I from the perspective of IBD alleles. Thus, there are two types of sibling B's IBD genotype with equal probabilities, $e_I x_I$ and $f_I x_I$.

Under H_p, the distribution of sibling B's IBD genotype can be derived as follows: (i) derive the IBD genotype distribution of sibling A's non-participated parent (labeled as NPA); (ii) derive the IBD genotype distribution of sibling B's non-participated parent (labeled as NPB); and (iii) derive sibling B's IBD genotype.

$$\begin{aligned}
\Pr\left(B \equiv \mathcal{G}_j \middle| A, P, H_p\right) &= \sum_x \left[\Pr\left(NPA \equiv \mathcal{G}_x \middle| A, P, H_p\right) \times \Pr\left(B \equiv \mathcal{G}_j \middle| NPA \equiv \mathcal{G}_x, A, P, H_p\right)\right] \\
\Pr\left(B \equiv \mathcal{G}_j \middle| NPA \equiv \mathcal{G}_x, A, P, H_p\right) &= \sum_y \left[\Pr\left(NPB \equiv \mathcal{G}_y \middle| NPA \equiv \mathcal{G}_x, A, P, H_p\right) \times \Pr\left(B \equiv \mathcal{G}_j \middle| NPB \equiv \mathcal{G}_y, NPA \equiv \mathcal{G}_x, A, P, H_p\right)\right]
\end{aligned} \tag{S7}$$

(i) If not considering mutation, the IBD genotype of NPA must be $a_I x_I$ or $b_I x_I$, the probabilities of which can be calculated with Bayes' rules:

$$\Pr\left(NPA \equiv a_I x_I \middle| A, P, H_p\right) = \frac{\Pr\left(NPA \equiv a_I x_I \middle| P, H_p\right) \times \Pr\left(A \middle| NPA \equiv a_I x_I, P, H_p\right)}{\Pr\left(NPA \equiv a_I x_I \middle| P, H_p\right) \times \Pr\left(A \middle| NPA \equiv a_I x_I, P, H_p\right) + \Pr\left(NPA \equiv b_I x_I \middle| P, H_p\right) \times \Pr\left(A \middle| NPA \equiv b_I x_I, P, H_p\right)} \tag{S8}$$

Where $\Pr\left(NPA \equiv a_I x_I \middle| P, H_p\right) = \Pr\left(NPA \equiv a_I x_I\right) = p_a$ under non-inbred assumption. And because of the same assumption, the allele b of sibling A must inherited from P, i.e., $\Pr\left(A \middle| NPA \equiv a_I x_I, P, H_p\right) \propto d_{Pb}$. Thus,

$$\begin{cases}
\Pr\left(NPA \equiv a_I x_I \middle| A, P, H_p\right) = \dfrac{p_a d_{Pb}}{p_a d_{Pb} + p_b d_{Pa}} \\
\Pr\left(NPA \equiv b_I x_I \middle| A, P, H_p\right) = \dfrac{p_b d_{Pa}}{p_a d_{Pb} + p_b d_{Pa}}
\end{cases} \tag{S9}$$

(ii) Under H_p, NPB should be unrelated to P and the relationship between him/her with sibling A should happened through NPA, i.e., the genotype of P and sibling A should be useless for the IBD genotype inference of NPB when the IBD genotype of

NPA is given, i.e., $\Pr\left(NPB \equiv \mathcal{G}_y \middle| NPA \equiv \mathcal{G}_x, A, P, H_p\right) = \Pr\left(NPB \equiv \mathcal{G}_y \middle| NPA \equiv \mathcal{G}_x, H_p\right)$. If no inbreeding factor is considered, the relationship between NPA and NPB can be described by $\kappa = \{\kappa_0, \kappa_1, \kappa_2\}$. It can be seen that, if $NPB \equiv a_I x_I$ as example, there can be 2 types of NPB's IBD genotype, $a_I x_I$ and $x_I y_I$, with probabilities of $\kappa_2 + \kappa_1/2 = 2\varphi$ and $\kappa_0 2 + \kappa_1/2 = (1 - 2\varphi)$, respectively, if φ denotes the kinship coefficient between NPA and NPB; Similarly, if $NPB \equiv b_I x_I$ as example, NPB's IBD genotype can be $b_I x_I$ or $x_I y_I$, with probabilities of 2φ and $(1 - 2\varphi)$, respectively;

(iii) Similar to the inference of NPB's IBD genotype, NPA and sibling A's genotypes are useless for the IBD genotype inference of sibling B when the IBD genotype of NPB and P are given, i.e.,

$$\Pr\left(B \equiv \mathcal{G}_j \middle| NPB \equiv \mathcal{G}_y, NPA \equiv \mathcal{G}_x, A, P, H_p\right) = \Pr\left(B \equiv \mathcal{G}_j \middle| NPB \equiv \mathcal{G}_y, A, H_p\right) \tag{S10}$$

In summary, there can be 6 types of sibling B's IBD genotype, the inference process is listed in Table S5:

Table S5: Sibling B's IBD genotype

B	NPA	$\Pr\left(NPA \equiv a_I x_I \middle| A, P, H_p\right)$	NPB	$\Pr\left(NPB \equiv \mathcal{G}_y \middle| NPA \equiv \mathcal{G}_x, H_p\right)$	$\Pr_1$*	Total Probability
$a_I e_I$	$a_I x_I$	$\frac{p_a d_{Pb}}{p_a d_{Pb} + p_b d_{Pa}}$	$a_I x_I$	2φ	$\frac{1}{4}$	$\frac{\varphi p_a d_{Pb}}{2 p_a d_{Pb} + 2 p_b d_{Pa}}$
$a_I f_I$	$a_I x_I$	$\frac{p_a d_{Pb}}{p_a d_{Pb} + p_b d_{Pa}}$	$a_I x_I$	2φ	$\frac{1}{4}$	$\frac{\varphi p_a d_{Pb}}{2 p_a d_{Pb} + 2 p_b d_{Pa}}$
$b_I e_I$	$b_I x_I$	$\frac{p_b d_{Pa}}{p_a d_{Pb} + p_b d_{Pa}}$	$b_I x_I$	2φ	$\frac{1}{4}$	$\frac{\varphi p_b d_{Pa}}{2 p_a d_{Pb} + 2 p_b d_{Pa}}$
$b_I f_I$	$b_I x_I$	$\frac{p_b d_{Pa}}{p_a d_{Pb} + p_b d_{Pa}}$	$b_I x_I$	2φ	$\frac{1}{4}$	$\frac{\varphi p_b d_{Pa}}{2 p_a d_{Pb} + 2 p_b d_{Pa}}$
$e_I x_I$	$a_I x_I$	$\frac{p_a d_{Pb}}{p_a d_{Pb} + p_b d_{Pa}}$	$a_I x_I$	2φ	$\frac{1}{4}$	$\frac{1-\varphi}{2}$
			$x_I y_I$	$1 - 2\varphi$	$\frac{1}{2}$	
	$b_I x_I$	$\frac{p_b d_{Pa}}{p_a d_{Pb} + p_b d_{Pa}}$	$b_I x_I$	2φ	$\frac{1}{4}$	
			$x_I y_I$	$1 - 2\varphi$	$\frac{1}{2}$	
$f_I x_I$	$a_I x_I$	$\frac{p_a d_{Pb}}{p_a d_{Pb} + p_b d_{Pa}}$	$a_I x_I$	2φ	$\frac{1}{4}$	$\frac{1-\varphi}{2}$
			$x_I y_I$	$1 - 2\varphi$	$\frac{1}{2}$	
	$b_I x_I$	$\frac{p_b d_{Pa}}{p_a d_{Pb} + p_b d_{Pa}}$	$b_I x_I$	2φ	$\frac{1}{4}$	
			$x_I y_I$	$1 - 2\varphi$	$\frac{1}{2}$	

* $\Pr_1 = \Pr\left(B \equiv \mathcal{G}_j \middle| NPB \equiv \mathcal{G}_y, A, H_p\right)$;

With calculation similar to Eq. (24) in the main text, it can be derived that

$$LR = \frac{\varphi p_a d_{Pb}\left(\mathbb{1}_{ac} d_{Pd} + \mathbb{1}_{ad} d_{Pc}\right) + \varphi p_b d_{Pa}\left(\mathbb{1}_{bc} d_{Pd} + \mathbb{1}_{bd} d_{Pc}\right)}{\left(d_{Pa} p_b + d_{Pb} p_a\right)\left(d_{Pc} p_d + d_{Pd} p_c\right)} + (1 - \varphi) \tag{S11}$$

According to the equation, the function '*LRhsip*()' is constructed.

Input arguments 7 input arguments are needed for the function

- **A**: A data frame of 2 columns containing the genetic information of individual As;
- **B**: A data frame of 2 columns containing the genetic information of individual Bs;
- **P**: A data frame of 2 columns containing the genetic information of the identical parents;
- **af**: A data frame similar to **af** in '*pairsimu*()' function;
- **rare**: The frequency of rare alleles, which is similar to **rare** in '*LRparas*()' function;
- **allelename**: The data type of **A**, **B** and **P** data frames, similar to **allelename** in '*LRparas*()' function;
- **phi**: The kinship coefficient φ between the non-participant parents of the two half-siblings under Hp, with a default set of 0.5, i.e., assuming that individual A is a full-sibling of individual B.

Output value A data frame of **ss** rows and 1 columns would be output, containing the $\log_{10}$LR of each case.

Detailed description The function is performed as Algorithm 7.

Algorithm 7: LRhsip()

1. Conduct formal checks on the 3 genotype data frames, and **stop** if any error exists;
2. Extract or calculate the $\boldsymbol{d}$ parameters, $\mathbb{1}$ parameters, and $\boldsymbol{p}$ parameters in Eq. (S11) with codes similar to corresponding ones in '*LRparas*()' function;
3. Calculate LR according to Eq. (S11);
4. Transfer error values, i.e., where no allele shared between P with A or B, into $(1 - \varphi)$;
5. Transfer the LR value into $\log_{10}$LR value.

Note that if there is no allele sharing between P with A or B, LR cannot be calculated directly according to Eq. (S11) due to the 0 value of the denominator. In this case, $(1 - \varphi)$ would be the output.

Example Two examples are given, simulating and calculating LR with $\varphi = 0.5$ for 10,000 A-B-P groups when Hp (A is full-sibling to B) and Hd (NPA is unrelated to NPB) in the formula is true, respectively.

```
# Construct pedi data.frames for two types of pedigrees
pedi1 <- data.frame(Person=c("F","M","A","B"),
                    Father=c("RI","RI","F","F"),
                    Mother=c("RI","RI","M","M"))
pedi2 <- data.frame(Person=c("M","A","B"),
                    Father=c("RI","RI","RI"),
                    Mother=c("RI","M","M"))
LR_1=LR_2=data.frame(Log10CLR=rep(0,10000))
for (i in 1:42) {
    # Simulate 10000 groups of A/B/P where A is full sibling of B
    Genotype1=pedisimu(af = FortytwoSTR$afmatrix[[i ]], ss = 10000,pedi = pedi1)
    LR_1=LR_1+LRhsip(A=Genotype1[,5:6],B=Genotype1[,7:8],P=Genotype1[,3:4],
                     af = FortytwoSTR$afmatrix[[i ]], rare=FortytwoSTR$rare[i ][1,1])
    # Simulate 10000 groups of A/B/P where A is half sibling of B, i.e., the true phi=0
    Genotype2=pedisimu(af = FortytwoSTR$afmatrix[[i ]], ss = 10000,pedi = pedi2)
    LR_2=LR_2+LRhsip(A=Genotype2[,3:4],B=Genotype2[,5:6],P=Genotype2[,1:2],
                     af = FortytwoSTR$afmatrix[[i ]], rare=FortytwoSTR$rare[i ][1,1])
  }
# histograms of CLR distributions in the two groups
xmin<-floor(min(min(LR_1$Log10CLR),min(LR_2$Log10CLR)))
xmax<-ceiling(max(max(LR_1$Log10CLR),max(LR_2$Log10CLR)))
par(mfrow = c(1, 2))
hist (LR_2$Log10CLR,xlab = expression(log[10]~CLR),main = "Fault pedigree",
      xlim = c(xmin,xmax), col = "red")
hist (LR_1$Log10CLR,xlab = expression(log[10]~CLR),main = "True cases",
      xlim = c(xmin,xmax), col = "blue")
```

4.3.8 '*LRgpgcam*()'

The function will compute the likelihood ratio for grandparentage determination using the reference of an uncle or aunt. In such scenarios, a child (C, with genotype *cd*) is claimed to be the grandchild of another individual (GP, with genotype *ef*), and the offspring (A, with genotype *ab*) of the alleged grandparent is involved. Additionally, the genetic information of B's other parent (M, with genotype *gh*) may or may not be available. Hp assumes that B is the offspring of A's full sibling, and Hd assumes that B is not related to GP and A. The consideration of inbreeding is not included.

Derivation of LR in the identification Similar to the derivation in '*LRhsip*()' function, C can be regarded as individual B in Eq. (21) of the main text and the relationship among other individuals would remain unchanged. Additionally, the genotype of GP and A is unrelated to C's IBD genotype under Hd. Thus,

$$LR = \frac{\sum_{j}\left[\Pr\left(C \equiv \mathcal{G}_j \middle| A, GP, M, H_p\right) \times \Pr\left(\mathcal{G}_j \equiv cd\right)\right]}{\sum_{k}\left[\Pr\left(C \equiv \mathcal{G}_k \middle| M, H_d\right) \times \Pr\left(\mathcal{G}_k \equiv cd\right)\right]} \tag{S12}$$

If label C's other parent (i.e, GP's offspring and A's full-sibling) as NP, $\Pr\left(C \equiv \mathcal{G}_j \middle| A, GP, M, H_p\right)$ can be calculated as follows. Note that the individuals useless in specific reference have been removed from corresponding probabilities.

$$\Pr\left(C \equiv \mathcal{G}_j \middle| A, GP, M, H_p\right) = \sum_{x}\left[\Pr\left(NP \equiv \mathcal{G}_x \middle| A, GP, H_p\right) \times \Pr\left(C \equiv \mathcal{G}_j \middle| NP \equiv \mathcal{G}_x, M, H_p\right)\right] \tag{S13}$$

It is clear that GP, A, and NP can be referred to as P, sibling A, and sibling B respectively in the context of identifying 4.3.7, and the distribution of NP's IBD genotype is illustrated in Table S5. Hence, there may be 10 or 5 variations of C's IBD genotypes, based on the presence or absence of M, and consequently, the LR can be computed as follows if M is available:

$$LR = \frac{d_{GPc}d_{Md} + d_{GPd}d_{Mc}}{4p_c d_{Md} + 4p_d d_{Mc}} + \frac{p_a d_{GPb}\left(\mathbb{1}_{ac}d_{Md} + \mathbb{1}_{ad}d_{Mc}\right) + p_b d_{GPa}\left(\mathbb{1}_{bc}d_{Md} + \mathbb{1}_{bd}d_{Mc}\right)}{4\left(p_a d_{GPb} + p_b d_{GPa}\right)\left(p_c d_{Md} + p_d d_{Mc}\right)} + \frac{1}{4} \tag{S14}$$

If M is unavailable,

$$LR = \frac{d_{GPc}}{8p_c} + \frac{d_{GPd}}{8p_d} + \frac{p_a d_{GPb} \mathbb{1}_{ac} + p_b d_{GPa} \mathbb{1}_{bc}}{8p_c\,(p_a d_{GPb} + p_b d_{GPa})} + \frac{p_a d_{GPb} \mathbb{1}_{ad} + p_b d_{GPa} \mathbb{1}_{bd}}{8p_d\,(p_a d_{GPb} + p_b d_{GPa})} + \frac{1}{4} \quad \text{(S15)}$$

It can be seen that the Eq. (S14) can be transferred into Eq. (S15) by replacing d_{Mc} and d_{Md} with p_c and p_d, respectively. According to the equation, the function '*LRgpgcam*()' is constructed.

Input arguments 7 input arguments are needed for the function

- **A**: A data frame of 2 columns containing the genetic information of individual As;
- **C**: A data frame of 2 columns containing the genetic information of individual Cs;
- **GP**: A data frame of 2 columns containing the genetic information of individual GPs;
- **M**: *NULL* or a data frame of 2 columns containing the genetic information of individual Ms;
- **af**: A data frame similar to **af** in '*pairsimu*()' function;
- **rare**: The frequency of rare alleles, which is similar to **rare** in '*LRparas*()' function;
- **allelename**: The data type of the 4 genotype data frames, similar to **allelename** in '*LRparas*()' function;

Output value A data frame of **ss** rows and 1 columns would be output, containing the $\log_{10}$LR of each case.

Detailed description The function is performed as Algorithm 8.

Algorithm 8: LRgpgcam()

Conduct formal checks on the 3 genotype data frames containing genotypes, and **stop** if any error exists;
Extract the 4 ***p*** values in Eq. (S14) with method similar to "#" in '*LRparas*()' function;
if $M \neq$ *'NULL'* **then**
    Conduct formal checks on **M** data frame, **stop** if error;
    Calculate d_{Mc} and d_{Md};
else
    Set $d_{Mc} = p_c$ and $d_{Md} = p_d$;
end
Calculate other ***d*** or $\mathbb{1}$ parameters;
Calculate the LR results according to Eq. (S14).

The three parts of Eq. (S14) correspond to the three scenarios of C's inheritance under Hp: i) C's paternal allele originated from one of GP's two alleles; ii) C's paternal allele is IBD to the allele NP passed to A ; and iii) C's paternal allele is IBD to the allele NP not passed to A. In the first two components, two factors appear within the denominator: $(p_a d_{GPb} + p_b d_{GPa})$ and $(p_c d_{Md} + p_d d_{Mc})$, which would equal 0 when there is no allele sharing between GP-A and M-C pairs, respectively. Such 0 values would occur due to mutation or the false relationship between the corresponding, and if so, the parts with 0 value in the denominator would be treated as 0 in the LR calculation equation. The output value of LR would have a minimum value of 1/4 because of part iii) in the formula.

Example Two examples are given, simulating and calculating CLR based on the 42 STRs in *FortytwoSTR* dataset, for 10,000 A-C-GP-P groups when Hp and Hd in the formula is true, respectively.

```
# Construct pedi data.frames for two types of pedigrees
pedi1 <- data.frame(Person=c("GF","GM","F","A","M","C"),
Father=c("RI","RI","GF","GF","RI","F"),
Mother=c("RI","RI","GM","GM","RI","M"))
pedi2 <- data.frame(Person=c("GF","GM","F","A","M","C"),
Father=c("RI","RI","RI","GF","RI","F"),
Mother=c("RI","RI","RI","GM","RI","M"))
LR_1=LR_2=data.frame(Log10CLR=rep(0,10000))
for (i in 1:42) {
  # Simulate 10000 group of pedigrees where the Hp is true
    Genotype <- pedisimu(af = FortytwoSTR$afmatrix[[i]], ss = 10000,pedi = pedi1)
    LR_1 <-LR_1+ LRgpgcam(A=Genotype[,7:8],C=Genotype[,11:12],GP=Genotype[,1:2],M=Genotype[,9:10],
                          af=FortytwoSTR$afmatrix[[i]], rare=FortytwoSTR$rare[i][1,1])
  #'#Simulate 10000 group of false pedigrees, i.e., P and C is unrelated to GP and A
  Genotype <- pedisimu(af = FortytwoSTR$afmatrix[[i]], ss = 10000,pedi = pedi2)
    LR_2 <-LR_2+ LRgpgcam(A=Genotype[,7:8],C=Genotype[,11:12],GP=Genotype[,1:2],M=Genotype[,9:10],
                          af=FortytwoSTR$afmatrix[[i]], rare=FortytwoSTR$rare[i][1,1])
  }
# histograms of CLR distributions in the two groups
xmin<-floor(min(min(LR_1$Log10CLR),min(LR_2$Log10CLR)))
xmax<-ceiling(max(max(LR_1$Log10CLR),max(LR_2$Log10CLR)))
par(mfrow = c(1, 2))
 hist (LR_2$Log10CLR,xlab = expression(log[10]~CLR),main = "Fault pedigree",
      xlim = c(xmin,xmax), col = "red")
 hist (LR_1$Log10CLR,xlab = expression(log[10]~CLR),main = "True cases",
      xlim = c(xmin,xmax), col = "blue")
```

4.3.9 ‘*logLR*()’

The function would calculate parameters for a single pairwise case, including CIBS score and multiple types of $\log_{10}$CLR.

Input arguments 8 input arguments are needed for the function

- **AB**: A data frame of 4 columns containing the genetic information of the two individuals, each two columns for each; Each row of the data frame contains the information of the four allele on a marker, the name of which should be set as the row names. Note that all the row names of this data frame should be included in the data frame names of list **afmatrix** and column names of data frame **rare**, otherwise, the function would report an error;
- **afmatrix**: *NULL* or a list of multiple data frames containing allele frequency data on multiple markers, which can be generated using 4.3.1 function. The data frame names should be marker names. For each data frame, there should be 1 column and multiple rows, the column name should be ’Freq’ and row names should be allele names on the corresponding marker.
- **rare**: *NULL* or a data frame of 1 row and multiple columns, containing frequency of rare alleles on each marker. The column names should be the name of each marker;
- **allelename**: The data type of the genotype data frame, similar to **allelename** in ‘*LRparas*()’ function;
- **stepPI**: Setting of mutation calculation method similar to **stepwisePI** in ‘*LRparas*()’ function;
- **adelta3**: Distributions of the IBD coefficient of multiple outbred H_p in LR calculation, which should be a data.frame with 3 columns and x rows, where x denote the number of such LRs being calculated. The names of columns should be ‘*k0*’, ‘*k1*’ and ‘*k2*’, and the names of rows should be the names of each LR. If no outbred LR is needed, set as *NULL*;
- **adelta9**: Distributions of the IBD coefficient of multiple inbred H_p in LR calculation, which can be input similar to argument‘**adelta3**’, and the column names should be ‘*D1*’ to ‘*D9*’. If no inbred LR is needed, set as *NULL*;
- **mu**: mutation rate in parentage testing, with a default of 0.002

Output value A list of 2 data frames would be output: (i) “results_on_each_marker”: a data frame containing calculation results on each marker, with *nl* rows and multiple columns, each row for a marker and each column for a type of parameter, including IBS and multiple log10 values of LRs; (ii) ”total_results_of_the_case”: a data frame of 1 column and multiple rows, containing the CIBS and log10CLR results for the whole case.

Detailed description The function is performed as Algorithm 9.

Algorithm 9: logLR()

1 Conduct formal checks on the input arguments, and **stop** if any error exists;
2 Calculate IBS score on each marker as the 1st column of “results_on_each_marker” data frame, with method similar to “‡” of ‘*LRparas*()’ function;
3 **if** ***adelta3***=***adelta9***=*NULL* **then**
4   Output “results_on_each_marker” directly;
5 **else**
6   Extract p_c and p_d with a for loop; /* † */
7   Calculate LRid and PInomu, as well as PImu according to **stepPI** setting, similar to ‘*LRparas*()’ function;
8   **if** ***adelta9***=*NULL* **then**
9   **for** *i=1:(row number of **adelta3**)* **do**
10   Calculate $\log_{10}$LR with LRid, PInomu or PImu, according to κ distribution of the ith row of **adelta3**;
11   Bind the result with “results_on_each_marker” data frame as the (i+1) column;
12   **end**
13   **else**
14   Extract or calculate p_a, $\mathbb{1}_{ab}$ and $\mathbb{1}_{cd}$;
15   Calculate the factors of $\Delta_1 \sim \Delta_6$ in Eq. (15) of the main text;
16   **if** ***adelta3***=*NULL* **then**
17   **for** *i=1:(row number of **adelta9**)* **do**
18   Calculate $\log_{10}$LR with the LRid, PInomu or PImu, and the 6 factors in line 15, according to Δ distribution of the ith row of **adelta9**;
19   Bind the result with “results_on_each_marker” data frame as the (i+1) column;
20   **end**
21   **else**
22   Calculate outbred and inbred $\log_{10}$LRs one type by one type with two for loops;
23   **end**
24   **end**
25 **end**
26 Construct ”total_results_of_the_case” data frame, by calculate total results of each parameters in “results_on_each_marker” data frame using “as.data.frame(colSums(...))” code;
27 Output the 2 data frames “results_on_each_marker” and ”total_results_of_the_case” as a list.

‡ Frequency data would be extracted differently from single locus functions such as ‘*LRparas*()’, as the frequency data are different among markers. “For()” loop would be needed and the code would differ according to **allelename** setting. Take p_c as example:

```
if (isTRUE(allelename)) {
  for (i in 1:nl) {
    para$pc[i]<-afmatrix[[rownames(AB)[i]]][as.character(AB[i,3]),]
    if(is.na(para$pc[i])){
      para$pc[i]<-rare[[rownames(AB)[i]]]
    }
  }
} else{
  for (i in 1:nl) {
    para$pc[i]<-afmatrix[[rownames(AB)[i]]][AB[i,3],]
    if(is.na(para$pc[i])){
      para$pc[i]<-rare[[rownames(AB)[i]]]
    }
  }
}
```

Example An example is given, simulating and calculating CLR for a pairwise case:

```
AB<-data.frame(a=rep(0,42),b=rep(0,42),c=rep(0,42),d=rep(0,42))
for (i in 1:42) {
    temp<-pairsimu(af = FortytwoSTR$afmatrix[[i]], ss = 1, delta = c(0,1,0), allelename = TRUE)
    AB[i,]=temp
    rownames(AB)[i]=names(FortytwoSTR$afmatrix)[i]
  }
adelta3<-data.frame(k0=c(0,0.25,0.5),k1=c(1,0.5,0.5),k2=c(0,0.25,0),row.names = c("PC","FS","HS"))
adelta9<-data.frame(D1=0,D2=0,D3=0,D4=0,D5=0.25,D6=0,D7=0.25,D8=0.5,D9=0,row.names = "FIMCpair")
results <-logLR(AB=AB,afmatrix=FortytwoSTR$afmatrix,rare=FortytwoSTR$rare,stepPI=TURE,adelta3=adelta3,adelta9=adelta9)
```

4.3.10 ‘*testsimulation*()’

An all-in-one simulating and calculating solution for kinship analysis: Combining the genotype generating and LR calculating functions, a integrated function is given for the batch simulation and calculation in kinship analysis, which can generate genotype combinations of multiple individual pairs with specific relationship on multiple independent markers and then calculate multiple types of CLR for each pair.

Input arguments 8 input arguments are needed for the function

- **afmatrix**: The allele frequency data utilized in the simulation and calculation, which is in a format similar to the **afmatrix** in the ‘*logLR*()’ function, with the exception that the argument cannot be *NULL*.
- **ss**: The sample size to be simulated;
- **tdelta**: The distribution of Δ or κ describing the true relation between the simulated individuals;
- **stepPI**: Setting of mutation calculation method similar to **stepwisePI** in ‘*LRparas*()’ function;
- **adelta3**: Similar to **adelta3** in ‘*logLR*()’ function;
- **adelta9**: Similar to **adelta9** in ‘*logLR*()’ function;
- **pedname**: The name of the simulated relationship;
- **mu**: mutation rate in parentage testing, with a default of 0.002.

Output value The output of this function will be a data.frame consisting of ss rows and multiple columns. Each row in the data.frame will correspond to the calculation results of a simulated pair defined by the following code. The first column will contain the relation name defined by the argument ‘*pedname*’, while the second column will contain the CIBS value for each pair. The remaining columns will contain the 10 logarithms of multiple types of LRs defined by the arguments ‘*adelta3*’ and ‘*adelta9*’.

Detailed description The function will conduct an idealized simulation using allele frequency of multiple markers. Typically, such simulations are useful for the initial assessment of a specific identification panel. Due to its primary nature, some confounding factors are disregarded in the coding of this function, such as excluding mutation factors when generating simulated individuals, which results in rare alleles not being generated and hence, the frequency of rare alleles does not need to be considered. The function is performed as Algorithm 10.

Algorithm 10: testsimulation()

1 Conduct formal check on the input delta values, and **stop** if any error exists;
2 Extract *nl* as the number of data frames contained in **afmatrix** list;
3 Construct the "result" data frame with *ss* rows and a number of columns determined by the **adelta3** and **adelta9** settings. Set all values in the data frame to 0;
4 **for** *i=1:nl* **do**
5 Generate genotypes for *ss* individual pairs according to the relationship described by **tdelta**, based on the frequency data contained in the ith data frame of the **afmatrix** list, using the '*pairsimu*()' function;
6 Calculate CIBS and multiple $\log_{10}$LR values according to the **adelta3**, **adelta9**, **stepPI**, and **mu** settings, using the '*LRparas*()' function;
7 Sum the calculation results with the updated "result" data frame;
8 **end**
9 Output the "result" data frame.

Example An example is given, simulating and calculating 4 types of CLRs for 10,000 parent-child pairs, based on the 42 STRs in *FortytwoSTR* dataset:

```
adelta3 <-data.frame(k0=c(0,0.25,0.5) ,k1=c (1,0.5,0.5) ,k2=c (0,0.25,0) ,row.names=c("PC","FS","HS"))
adelta9 <-data.frame(D1=0,D2=0,D3=0,D4=0,D5=0.25,D6=0,D7=0.25,D8=0.5,D9=0,row.names="FIMCpair")
data(FortytwoSTR)
results <- testsimulation ( afmatrix =FortytwoSTR[["afmatrix" ]], ss=10000, tdelta =c (0,1,0) , adelta3 =adelta3 , adelta9 =adelta9 ,pedname="PC")
results $ total _ results _of_the_case
```

4.3.11 '*outputCSV*()'

The function would output population data such as "*FortytwoSTR*" into .csv files, which can be used for '*EvaluatePanel*()' function.

Input arguments 2 input arguments are needed for the function

- **data**: The name of a list of 4 data frames in format similar to "*FortytwoSTR*";
- **strpath**: The pathway to output the resulting .csv file;

Output value A .csv file in ISFG format, i.e., put allele frequency data in the cells right and down to B2

Detailed description The function is performed as Algorithm 11.

Algorithm 11: outputCSV()

1 Conduct formal check on the input delta values, and **stop** if the data list is not in format similar to *FortytwoSTR*;
2 Extract *nl* as the number of data frames contained in 'afmatrix' list;
3 Extract allele names into a one-column data frame 'allelenames' from the row names of the data frames contained in 'afmatrix' list;
4 Remove the duplicate in 'allelenames' data frame and sort it from small to large, set the row number of 'allelenames' as *maxa*;
5 Construct a data frame "results" with (1+nl) columns and (1+maxa) rows, set column names as the marker names, data in the 1st column as 'allelenames'; **for** *i=1:nl* **do**
6 Extract the allele frequencies on the ith marker into the (i+1)th column of 'results' data frame and replace the NA values with 0;
7 **end**
8 Extract the number of individual on each marker into the last row; Outout the result data frame

Example An example is given, outputting the *FortytwoSTR* data into a .csv file:

```
path<-tempdir()
outputCSV(FortytwoSTR,file.path(path,'data.csv'))
```

4.4 Data involved

4.4.1 *FortytwoSTR*

A list of 4 data frames, containing the allele frequency data for the Chinese Han population on a 42-plex STR panel, along with forensic parameters. The list is created using the '*EvaluatePanel*()' function with the default settings, which are: (i) utilizing the values in the last row as the sample size in the population survey; (ii) computing frequencies of rare alleles using the 'ISFG' method.

```
FortytwoSTR<-EvaluatePanel(strpath = "https://raw.githubusercontent.com/Guanju-Ma/data111/main/42STR.csv")
```

4.4.2 *pediexample*

A data frame containing an example of the input form of **pedi** argument used in ‘*pedisimu*()’ function, which is generated with the following code:

```
pediexample<-data.frame(Person=c("GF","GM","F1","F2","M1","M2","A","B"),
  Father=c("RI","RI","GF","GF","RI","RI","F1","F2"),
  Mother=c("RI","RI","GM","GM","RI","RI","M1","M2"))
```

which results in a data frame as follows:

	Person	Father	Mother
##1	“GF”	“RI”	“RI”
##2	“GM”	“RI”	“RI”
##3	“F1”	“GF”	“GM”
##4	“F2”	“GF”	“GM”
##5	“M1”	“RI”	“RI”
##6	“M2”	“RI”	“RI”
##7	“A”	“F1”	“M1”
##8	“B”	“F2”	“M2”

5 Evaluation of R package *KINSIMU*

Multiple types of evaluation were taken on the function ‘‘*testsimulation*()’’ based on allele frequency data on 42 autosomal STR markers in Chinese Han population, which is given as data ‘*FortitwoSTR*’ in the package. In the following parts, the simulation of a type of identification would be carried out by simulating *ss* pairs of specific type of relatives, as well as equal number of unrelated pairs. Different types of CLR would be calculated for each pair, including cumulative paternity index (CPI, $\kappa = \{0, 1, 0\}$ under H_p) , cumulative full-sibling index (CFSI, $\kappa = \{0.25, 0.5, 0.25\}$ under H_p), cumulative half-sibling index (CHSI, $\kappa = \{0.5, 0.5, 0\}$ under H_p) and cumulative first-cousin index (CFCI, $\kappa = \{0.25, 0.75, 0\}$ under H_p). All simulation were taken placed on a same personal device with Intel® Core™ i7-9700F CPU and 16GB RAM. Codes used in each type of evaluation are provided in File S3.

5.1 Stability

3rd-degree relationship identification was used for the evaluation of stability of the simulation code, based on all the 42 STRs with sample sizes being 10^4, 10^5, 10^6, and 10^7. Simulations of each *ss* were taken place for 100 times, CFCI and CIBS are calculated for each case, and comparisons among each time of simulation with same conditions (*ss* and the true relationship) are taken place. As shown in Figure S1 and Table S6 of this supplemental file, the simulation results would be more and more stable with the increase of sample size.

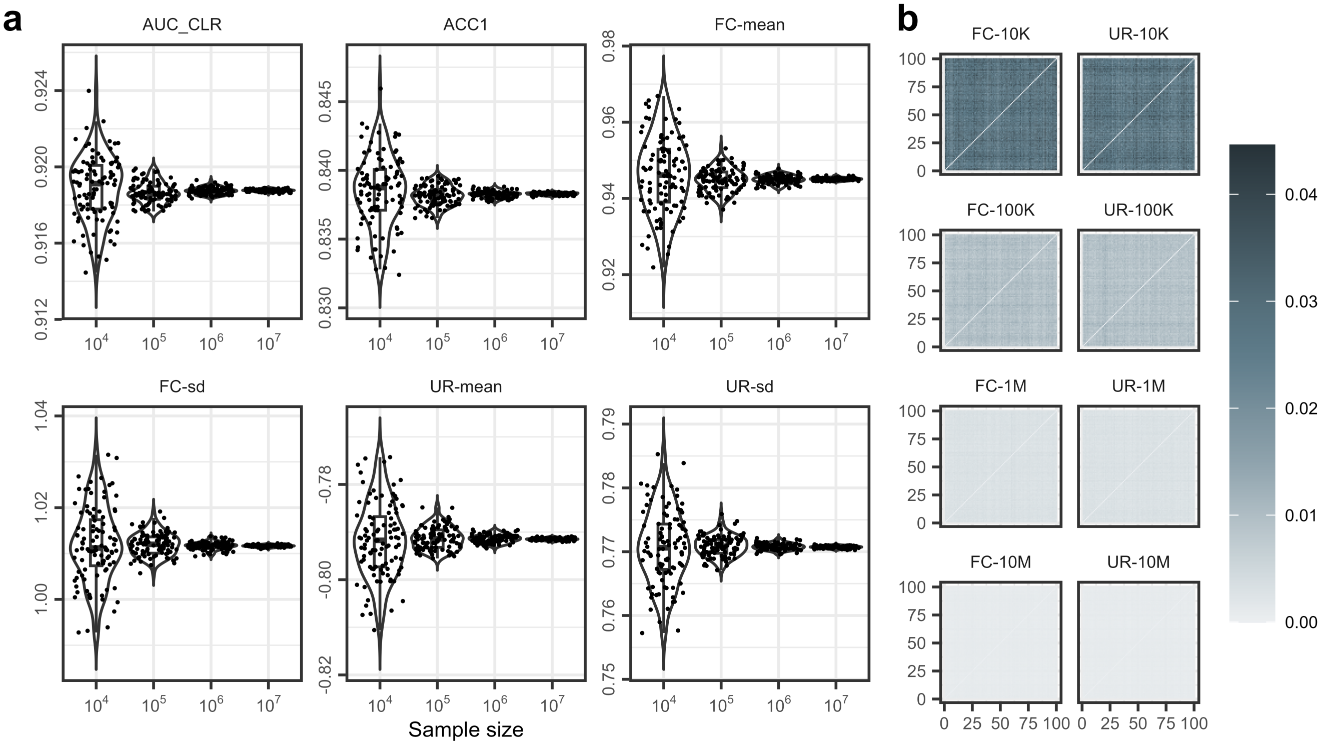

Figure S1: Results of stability testing. a) parameters obtained with CLR method for each simulation: i) AUC_CLR: AUC based on CLR distributions of pairs with two relationships; ii) ACC1: accuracy when CLR=1 is set as threshold to distinguish the two relationships; iii) mean and sd: Mean and standard deviation of CLR distributions of pairs with two relationships: FC and UR as first cousin and unrelated, respectively; b) Heatmap of the Hellinger distance (HD) of the CIBS distribution obtained from 100 simulations with other conditions being same, the maximum values of each group are listed above each subfigure.

Table S6: main indicators of the stability of the simulations

Indicator*	Relationship	Value under different sample size			
		10,000	100,000	1,000,000	10,000,000
CV of AUC_CLR	FC vs. UR	1.85×10^{-3}	5.92×10^{-4}	2.04×10^{-4}	7.22×10^{-5}
CV of ACC1	FC vs. UR	3.08×10^{-3}	8.42×10^{-4}	2.87×10^{-4}	1.03×10^{-4}
CV of the mean of LR	FC	1.04×10^{-2}	3.18×10^{-3}	1.12×10^{-3}	3.56×10^{-4}
	UR	-9.71×10^{-3}	-2.93×10^{-3}	-1.07×10^{-3}	-3.02×10^{-4}
CV of the sd of LR	FC	8.08×10^{-3}	2.45×10^{-3}	8.11×10^{-4}	2.23×10^{-4}
	UR	7.42×10^{-3}	2.09×10^{-3}	6.65×10^{-4}	2.49×10^{-4}
maximum HD	FC	0.0446	0.0142	0.0045	0.0015
	UR	0.0415	0.0137	0.0045	0.0014

* CV: coefficient of variation; AUC_CLR: AUC based on the CLR distribution of the two types of individual pairs; ACC1: the accuracy when CLR=1 is set as the threshold; sd: standard deviation; HD: hellinger distance; FC: first cousins; UR: unrelated pairs.

5.2 Inter-platform similarity

Simulations are taken place applying *KINSIMU* or *Familas* 3.3 based on the 42 STRs. 3 types of identification are taken as examples: a) full-sibling testing, labeled as FS vs. UR; b) half-sibling testing, labeled as HS vs. UR, c) first cousin testing, labeled as FC vs. UR. The sample size is set as 10,000, because with an ss higher than that, the *Familas* software will report an error due to out of memory. Results of the 6 simulations are listed in Figure S2, it can be seen that under the premise of other conditions being the same, the distributions of the results obtained by the two platforms are similar, and no significant difference was found after Wilcoxon rank sum test. The running times of our tool were 0.28 sec, 0.25 sec and 0.27 sec for group a), b) and c), respectively, and the corresponding ones of *Familias*3.3 were 22.54 sec, 20.69 sec and 198.52 sec, respectively. In other words, the tools described in this paper increases the simulation efficiency by at least 80 times.

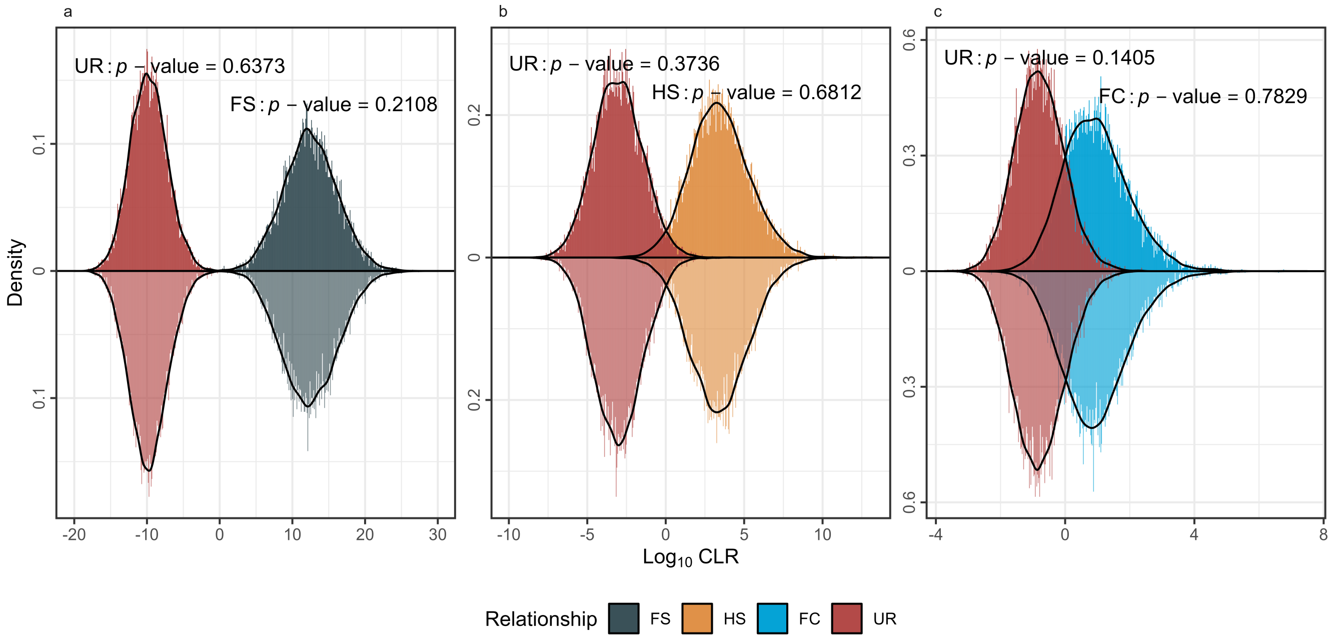

Figure S2: Comparison between *KINSIMU* with *Familas*. Distribution of three types of $Log_{10}CLR$ values simulated by *KINSIMU* (upper part) or *Familas* (bottom part): a) $Log_{10}CFSI$ in full-sibling identification; b) $Log_{10}CHSI$ in half-sibling identification; and c) $Log_{10}CFCI$ in first cousin identification. The *p*-values of Wilcoxon sum rank test for each group between the results of the two tools are listed in the figure. FS: full-siblings; HS: half-siblings; FC: first cousin; UR: unrelated pairs.

5.3 Inner-platform similarity

Genotypes on 42 STRs for different number (10^4, 10^5, 10^6 or 10^7, 50 times for each) of full-sibling pairs were simulated on a same device, applying function ''*testsimulation*()'' in *KINSIMU* or function '*sim*()' in '*relSim*', another R package designed for simulation. With *relSim*, three parameters are calculated for each pair, CIBS, CPI and CFSI, which are all calculated in our tool with the adjust of parameter 'adelta3'. The results are shown in Figure S3, showing that the simulations with the two packages were similar to each other, with the execution time of using *KINSIMU* being approximately one-tenth of that when using *relSim*.

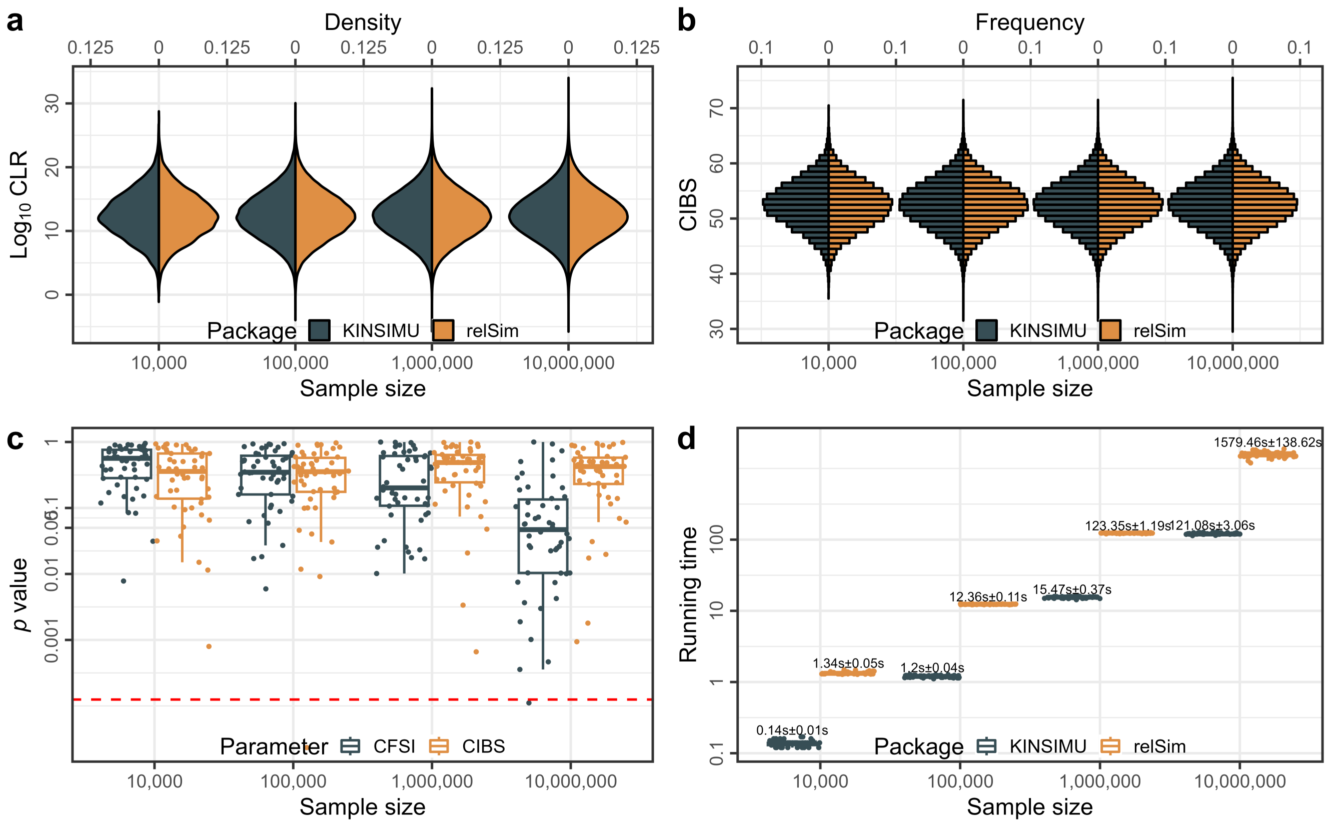

Figure S3: Comparison between *KINSIMU* with *relSim*. a) and b): Examples of the CFSI and CIBS distributions for the 4 types of sample size applying the two packages; c): *p*-values of Wilcoxon sum rank test for each simulation between the two packages, significant difference was found in 2 of the 400 simulations after Bonferroni correction (α=0.05/400, shown as the red dotted line); d): Running time comparison between the two packages.

5.4 Running speed estimation

144 types of simulations were performed from the following dimensions, to detailly assess factors that may have an impact on running time: **Identification** i) full-sibling identification, ii) 2nd degree kinship identification and iii) 3rd degree kinship identification; **Number of outputting parameters** i) Outputting only CIBS for each case; ii) Outputting CIBS and the CLR with Hp being the relationship of kinship pairs in each type of simulation; iii) Outputting CIBS, CLR and CPI, with mutation rate as PI when there was no sharing allele between the participants; iv) Outputting CIBS, CLR and CPI, considering empirical decreasing model of STR loci when calculating PI and there was no sharing allele between the participants; **Number of loci per case**: 14, 28 loci randomly selected from the 42 STRs, or all the 42 STRs; **Sample size**: 10^4, 10^5, 10^6 or 10^7 pairs of individuals with specific relationship vs. equal number of unrelated pairs. Each type of simulation was repeated for 5 times and the average speed of simulation was then calculated with the following equation for each type: $Speed = (sample\ size \times loci\ per\ case) \big/ (average\ running\ time)$. As shown in Figure S4, the impact of number of loci per case and the true relationship between kinship pairs of the identification on the average speed of simulation would be less than that of sample size and the type of output parameters. Roughly speaking, the average speed of simulation by function ''*testsimulation*()'' on our device would be 6,000,000 loci per second when only CIBS was calculated, 5,000,000 loci per second when CLRs without considering empirical decreasing model of STR mutation, and 2,000,000 loci per second if such model was considered.

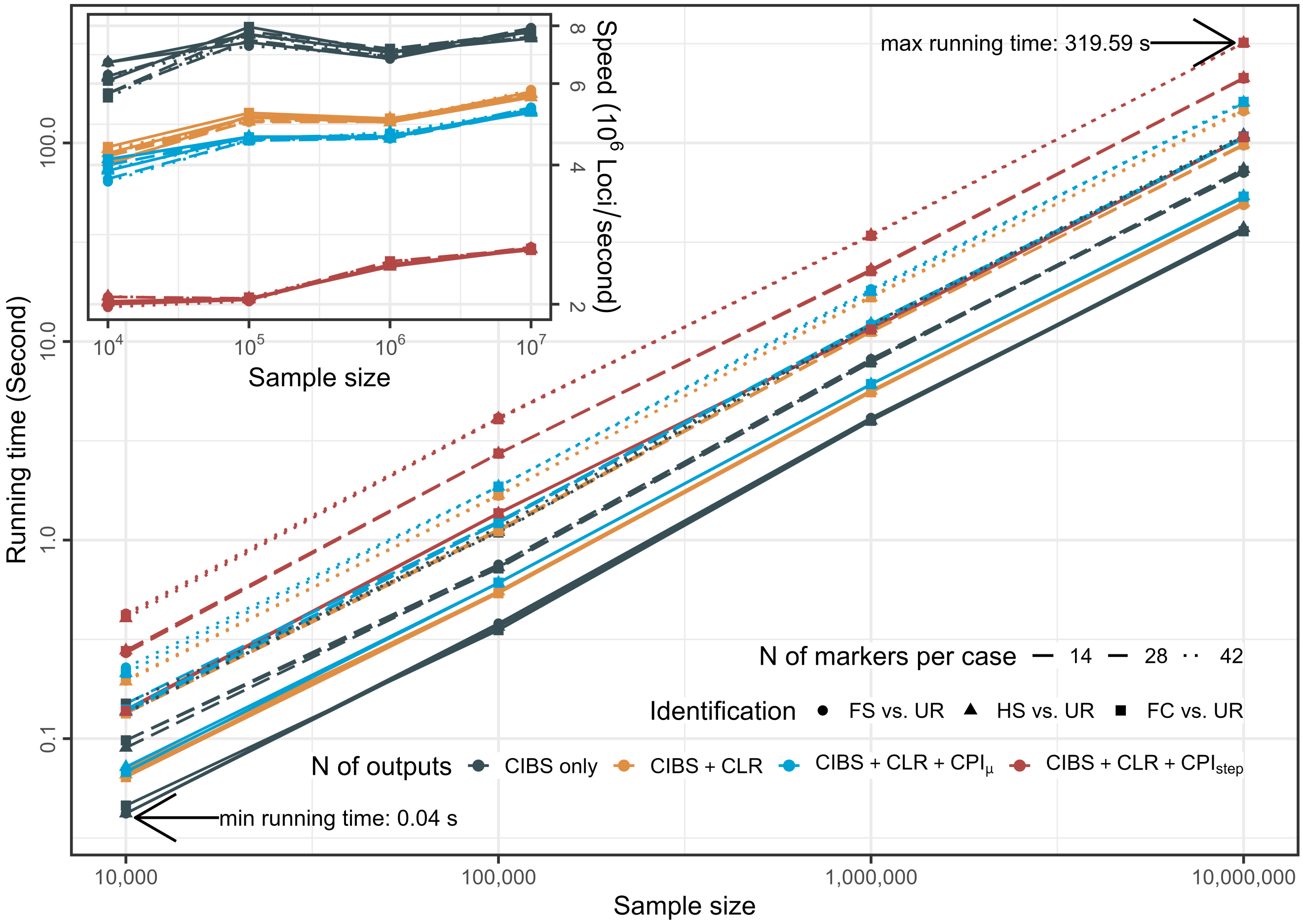

Figure S4: Average running time of different simulations. Averages of 5 repeats for each of the 144 types of simulation, the average speeds of simulation are listed in the left-top subfigure. FS: full-sibling; HS: half-sibling; FC: first cousin; UR: unrelated; CLR: the cumulative likelihood ratio with the relationship between kinship pairs simulated as Hp; CPI_μ: CPI without considering empirical decreasing model of STR mutation; CPI_{step}: CPI considering such model.

6 Comparison between the PI calculation codes of *KINSIMU* and *relSim*

This is a comparison between the basic calculation codes of the two packages *KINSIMU* and *relSim*, with the identical settings: 42 STRs, ss=1,000,000, the true relationship is parent-child, and only PI not considering mutation is calculated.

6.1 *KINSIMU* code

```
1   data(FortytwoSTR)
2   PI<-data.frame(PI=c(0,1000000))
3   for (i in 1:nl){
4     AB<-pairsimu(af = FortytwoSTR$afmatrix[[i]], ss=1000000,delta=c(0,1,0) , allelename=FALSE)
5     dc<-as.double(AB[,1]==AB[,3])+as.double(AB[,2]==AB[,3])
6     dd<-as.double(AB[,1]==AB[,4])+as.double(AB[,2]==AB[,4])
7     pc<-FortytwoSTR$afmatrix[[i]][AB[,3],]
8     pd<-FortytwoSTR$afmatrix[[i]][AB[,4],]
9     PI<-PI+log10(dc/(4*pc)+dd/(4*pd))
10  }
```

Notes: i) pairsimu() function would generate genotype of all cases on a single marker, i.e., generate 4000000 allele per loop in this example;

ii) LR are calculated per locus with unified calculation used in LRparas() function (line 5-9).

6.2 *relSim* code

```
Freq<-readFreqs('42STR.csv')
lrpc<-data.frame(lrPC=rep(1,1000000)))
for (block in 1:100){
  AB<-randomPCPairs(Freqs<-Freq,Blocksize=10000)
  for(i in 1:10000){
    i1 = (block-1)*10000 + i
    p1 = AB[[i]]$prof1
    p2 = AB[[i]]$prof2
    nLoc = 0
    while (lrpc[i1]>0 && nLoc<42) {
      markerfreq<-as.numeric(Freq$freqs[[nLoc]])
      parent<-p1[(2*nLoc+1):(2*nLoc+2)]
      child<-p2[(2*nLoc+1):(2*nLoc+2)]
      pc<-markerfreq[child[1]]
      if (child[2]==child[1]){
        if (parent[1]==parent[2]){
          if(parent[1]==child[1]) {
            lrpc[i1]=lrpc[i1]/pc
          } else {
            lrpc[i1]=0
          }
        } else {
          if (parent[1]!=child[1] && parent[2]!=child[1]){
            lrpc[i1]=0
          } else {
            lrpc[i1]=lrpc[i1]/(2*pc)
          }
        }
      } else {
        pd<-markerfreq[child[2]]
        if (parent[1]==parent[2]) {
          if (parent[1]==child[1]) {
            lrpc[i1]=lrpc[i1]/(2*pc)
          } else if (parent[1]==child[2]) {
            lrpc[i1]=lrpc[i1]/(2*pd)
          } else {
            lrpc[i1]=0
          }
        } else {
          if (parent[1]==child[1] && parent[2]==child[2]) {
            lrpc[i1]=lrpc[i1]*(pc+pd)/(4*pc*pd)
          } else if (parent[1]==child[1] || parent[2]==child[1]){
            lrpc[i1]=lrpc[i1]/(4*pc)
          } else if (parent[1]==child[2] || parent[2]==child[2]){
            lrpc[i1]=lrpc[i1]/(2*pd)
          } else {
            lrpc[i1]=0
          }
        }
      }
    }
  }
}
```

Note: i) The randomPCPair() function would return a list of 'Blocksize' vectors, each containing the genotype information of a PC pair. Such a generation process would be cut into 100 Blocks with the default set, i.e., generate 1680000 allele per loop in this example;

ii) Genotypes of the individual pairs are needed to be extracted case by case after being generated (line 8-9);
iii) LR are calculated per locus per case with listed calculation used in lrPC() function (line 14-50);
iv) The calculation of CPI would be cut once any 0 value occurs, which would save the performing time on some level in false pedigrees.
v) It should be noted that PI for situations under which the two individuals are identical heterozygotes but hold different order of alleles, e.g., 5/7-7/5, would be calculated with wrong formula as discussed in the main text. This would not effect the calculation for individual pairs generated using randomPCPair() function as it would reorder the genotype.
